# Neurocardiology: Brain–Heart Interactions in the Heart

**DOI:** 10.1002/mco2.70935

**Published:** 2026-09-03

**Authors:** Wentao Wang, Hao Jia, Han Han, Jiangping Song

**Affiliations:** ^1^ Department of Cardiac Surgery National Center for Cardiovascular Diseases Fuwai Hospital Chinese Academy of Medical Sciences and Peking Union Medical College Beijing China; ^2^ Beijing Key Laboratory of Xenotransplantation Animal Experimental Centre National Centre for Cardiovascular Disease Department of Cardiac Surgery National Center for Cardiovascular Diseases Fuwai Hospital Chinese Academy of Medical Sciences and Peking Union Medical College Beijing China; ^3^ State Key Laboratory of Cardiovascular Disease National Center for Cardiovascular Diseases Fuwai Hospital Chinese Academy of Medical Sciences and Peking Union Medical College Beijing China; ^4^ Department of Cardiac Surgery Fuwai Yunnan Hospital Chinese Academy of Medical Sciences Affiliated Cardiovascular Hospital of Kunming Medical University Kunming China; ^5^ Shenzhen Key Laboratory of Cardiovascular Disease Fuwai Hospital Chinese Academy of Medical Sciences Shenzhen China

**Keywords:** cardiac dementia, epileptic heart, heart failure, heart–brain interactions, psychocardiology, stroke–heart syndrome, Takotsubo syndrome

## Abstract

The heart and brain maintain systemic homeostasis through continuous bidirectional communication mediated by neural regulation, biochemical signaling, and mechanical transduction. Disruption of this communication is increasingly recognized as a contributor to cardiovascular, neurological, cognitive, and psychological disorders. Growing evidence indicates that autonomic imbalance, neuroimmune activation, hemodynamic disturbances, inflammation, altered interoception, and brain network remodeling can mediate cross‐organ injury between the heart and brain. These mechanisms underlie a spectrum of heart–brain comorbidities, but their shared pathways, disease‐specific manifestations, and integrated management strategies remain insufficiently understood. In this review, we summarize the physiological basis of heart–brain interactions, focusing on neural, biochemical, and mechanical pathways. We then discuss six representative disorders: Takotsubo syndrome, epileptic heart, stroke–heart syndrome, heart failure, cardiac dementia, and psychocardiology. For each condition, we highlight key epidemiological features, pathophysiological mechanisms, and integrated heart–brain management strategies. We also review clinical trials targeting heart–brain interactions, including autonomic modulation, biomarker monitoring, neuroimaging assessment, exercise therapy, psychological intervention, and pharmacological therapy. By integrating basic mechanisms, clinical phenotypes, and translational advances, this review provides a systematic framework for understanding heart–brain comorbidities and advancing precision heart–brain medicine.

## Graphical abstract

The heart and brain maintain systemic homeostasis through bidirectional communication involving neural, biochemical, and mechanical pathways. Dysregulation of these pathways contributes to a spectrum of conditions involving heart–brain interactions, including Takotsubo syndrome, the epileptic heart, stroke–heart syndrome, heart failure, cardiac dementia, and psychocardiological conditions. This review summarizes their epidemiological features, shared and disease‐specific pathophysiological mechanisms, and integrated management strategies. A better understanding of these interconnected pathways may facilitate earlier risk identification and support mechanism‐guided prevention, monitoring, and treatment of conditions involving heart–brain interactions.

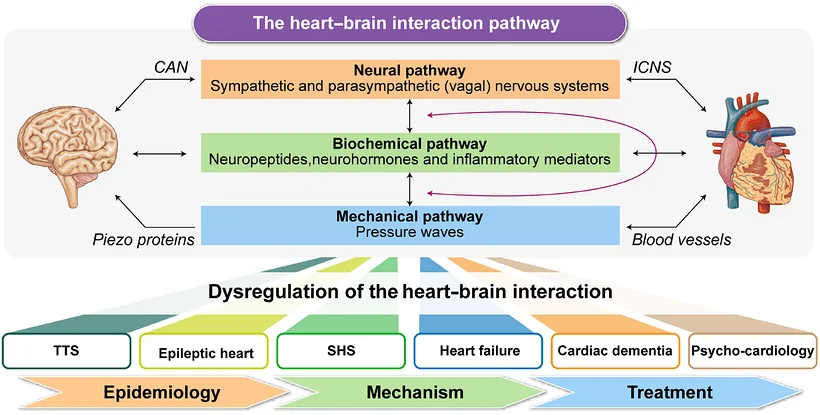

## 1 Introduction

Data from the Global Burden of Diseases, Injuries, and Risk Factors Study (GBD) 2021 identify heart–brain diseases as major contributors to the global burden of disease [1]. Specifically, ischemic heart disease and stroke accounted for 188 million and 160 million disability‐adjusted life years in 2021, respectively, ranking among the highest of all diseases globally [1]. Mental disorders also represent an increasing public health challenge. For instance, anxiety disorders and depression showed some of the most rapid increases in age‐standardized disability‐adjusted life‐year rates from 2010 to 2021, rising by 16.7 and 16.4%, respectively [1].

Research on heart–brain interactions originated from clinical observations of the frequent comorbidity between cardiac and cerebral disorders. In 1947, Byer and colleagues provided an early clinical description of this link, reporting associations between myocardial injury, arrhythmia, and cerebrovascular disease [2]. Traditionally, neurohumoral regulation was considered the primary physiological basis of heart–brain interactions. In this model, higher brain centers, such as the prefrontal cortex and limbic system, regulate cardiac function via the brainstem and autonomic nervous system (ANS). In return, the heart modulates central neural activity through peripheral receptors and supports cerebral function by delivering oxygen, nutrients, and hormonal signals via the circulation. However, recent evidence has reframed these interactions as the integrated action of neural, mechanical, and biochemical pathways [3]. These pathways are defined as follows: ① the neural pathway, which facilitates signal transduction between the central autonomic network (CAN) and the peripheral ANS; ② the mechanical pathway, which uses mechanoreceptors (e.g., PIEZO channels) to detect and transduce hemodynamic forces such as blood pressure (BP); and ③ the biochemical pathway, which involves endogenous bioactive molecules that regulate both neurological and cardiovascular function.

Subsequently, the clinical significance of heart–brain interactions has received increasing attention. This is exemplified by heart failure (HF) guidelines that recommend the management of cognitive impairment (CI) and depression, highlighting the heart's impact on the brain [4]. Conversely, the high prevalence of arrhythmias and other cardiovascular complications after stroke or in patients with epilepsy underscores the brain's influence on cardiac activity [5, 6]. Furthermore, autonomic dysfunction and impaired heart–brain communication are now recognized across a broad spectrum of conditions, including cardiac disorders (e.g., arrhythmias, HF) and neurological diseases (e.g., epilepsy, stroke, neurodegenerative disorders). At the population level, a large‐scale magnetic resonance imaging (MRI) study involving over 40,000 individuals revealed significant associations between cardiac traits and both white matter integrity and default mode network connectivity [7]. Genome‐wide association studies have also identified 80 genetic loci with pleiotropic effects on the heart and brain, establishing the first multidimensional genetic framework for heart–brain interactions [7]. Mechanistically, Huynh et al. recently demonstrated that myocardial infarction (MI) triggers a neuroimmune response that promotes sleep to limit cardiac inflammation, whereas sleep fragmentation exacerbates cardiac injury via the sympathetic–immune axis [8]. These findings provides a rationale for sleep‐focused interventions in cardiovascular disease (CVD).

Under pathological stress, heart–brain interactions are crucial for maintaining systemic homeostasis [8]. When dysregulated, these interactions can trigger pathological changes and create a vicious cycle that exacerbates heart–brain dysfunction, thereby promoting the onset of cardiovascular or neurological diseases or accelerating the progression of existing conditions. Although significant progress has been made in imaging, genetics, and neuroimmunology in recent years, a systematic and integrated summary of these complex mechanisms across different heart–brain comorbidities is still lacking. More importantly, targeted therapies aimed at heart–brain interactions remain limited, and there is an urgent clinical need to translate these findings into effective interventions.

This review summarizes the physiological basis of heart–brain interactions and provides a systematic overview of six representative heart–brain conditions, including Takotsubo syndrome (TTS), epileptic heart, stroke–heart syndrome (SHS), HF, cardiac dementia, and psychocardiology. It focuses on recent advances in epidemiology, pathophysiological mechanisms, and integrated heart–brain management.

## 2 Physiological Basis of Heart–Brain Interactions

Heart–brain interactions are mediated mainly through three pathways: neural, biochemical, and mechanical [3]. The neural pathway links the intrinsic cardiac nervous system (ICNS) and the CAN via the ANS, thereby regulating cardiac autonomic tone. The biochemical pathway involves diverse endogenous bioactive molecules—such as hormones, neuropeptide Y (NPY), and mediators of acute and chronic inflammation—that regulate cerebral and cardiovascular functions and autonomic tone. The mechanical pathway relies on mechanoreceptors (e.g., PIEZO channels) to sense and transduce hemodynamic signals, such as fluctuations in BP, arising from the peripheral and cerebral vasculature.

### 2.1 The Neural Pathway

The neural pathway serves as the most rapid communication channel in heart–brain interactions. Higher brain centers, including the frontal lobe and limbic system, process emotional, stress, and homeostatic signals. These signals are then relayed via descending brainstem pathways to the ANS, thereby precisely regulating cardiac function. Conversely, the heart reflexively modulates central neural activity through peripheral receptors. Thus, cardiomyocyte electrophysiology and contractility are governed by both intrinsic properties and extrinsic autonomic input relayed through the cardiac plexus [9].

#### 2.1.1 The CAN: Brain Regions Regulating the Heart

The CAN is an integrated system of interconnected brain regions that spans the neuroaxis, from the forebrain to the spinal cord [3]. Key components include the medial prefrontal, orbitofrontal, and cingulate cortices; insula; striatum; amygdala; hippocampus; thalamus; and hypothalamus, including the paraventricular nucleus (PVN). Within the brainstem, critical regions include the midbrain periaqueductal gray; the pons, including the locus coeruleus and parabrachial nucleus; and the medulla oblongata, including the caudal ventrolateral medulla (CVLM) and rostral ventrolateral medulla (RVLM), nucleus tractus solitarius (NTS), nucleus ambiguus, dorsal motor nucleus of the vagus, and area postrema. Functionally, the CAN integrates sensory, emotional, cognitive, and respiratory inputs to regulate sympathetic and parasympathetic outflow. It also receives and processes afferent signals from the heart, baroreceptors, and chemoreceptors [5].

Within the cerebral cortex, higher‐order centers—including the medial prefrontal, orbitofrontal, and cingulate cortices, coordinate autonomic functions linked to emotion and cognition and exert voluntary control over cardiovascular physiology [10]. The medial prefrontal cortex, for instance, is integral to emotional processing and stress‐related reflexes [11]. Notably, the left and right ventromedial prefrontal cortices facilitate parasympathetic activation and sympathetic inhibition, respectively [12]. Consequently, lesions of the right ventromedial prefrontal cortex cause paradoxical sympathetic disinhibition, promoting hyperexcitability that increases the risk of anxiety, hypertension, and arrhythmias [12]. Consistently, animal studies show that inhibiting glutamatergic transmission in the murine infralimbic cortex exacerbates chronic stress‐induced cardiovascular responses, including elevated BP, vascular dysfunction, and cardiac fibrosis [13]. These findings underscore prefrontal dysfunction as a key mechanism in stress‐induced cardiovascular pathology. In a seminal study using a rat heart rate (HR) biofeedback model, Yoshimoto and colleagues delineated the complete volitional pathway from the anterior cingulate cortex to the heart: anterior cingulate cortex → ventromedial thalamus → dorsomedial hypothalamus → nucleus ambiguus [14]. They identified theta‐frequency activity in anterior cingulate cortex neurons projecting to the ventromedial thalamus as crucial for triggering bradycardia. This top‐down voluntary mechanism induced sustained, training‐induced bradycardia accompanied by anxiolytic effects [14].

The insular cortex, a key limbic structure, is functionally segregated into a dorsocaudal region that integrates visceral and somatic sensations and an anteroventral region that is connected to the anterior cingulate cortex and amygdala and participates in emotion‐related autonomic regulation [9]. Consequently, the insular cortex is considered central to heartbeat perception, a core aspect of interoception and self‐awareness [15, 16]. Hsueh et al. employed an optogenetic cardiac pacemaker to achieve precise HR control in freely behaving mice [17]. Their study demonstrated that context‐specific tachycardia, at 900 bpm markedly exacerbated anxiety‐like behaviors in a risk‐associated context, but not in a safe setting, illustrating a bidirectional interplay between cardiac state and emotion [17]. Further investigations identified the posterior insular cortex as a critical hub for integrating cardiac signals and regulating anxiety, as its inhibition alleviated tachycardia‐induced anxiety [17]. This finding supports a causal role for peripheral physiological signals in shaping central emotional processing. Moreover, clinical observations in epilepsy patients reveal that left insular cortex stimulation induces bradycardia and hypotension, whereas right insular stimulation triggers a sympathetic excitatory response [18]. These observations suggest functional lateralization, whereby the left insula predominantly mediates parasympathetic control and the right insula cortex primarily governs sympathetic regulation.

The hypothalamus regulates key autonomic functions, including the regulation of HR and BP, thermoregulation, and autonomic responses to stress, emotion, and other physiological stimuli. The PVN, a critical hypothalamic component, is a central regulator of the hypothalamic–pituitary–adrenal (HPA) axis. The PVN also integrates cardiac and renal inputs to modulate sympathetic outflow and neuroendocrine function. In hypertension and HF, reduced neuronal nitric oxide synthase expression in the PVN impairs volume reflex mechanisms, thereby promoting sodium and fluid retention [19].

As a key limbic structure, the amygdala mediates cardiovascular responses to fear and anxiety. A cohort study of 498 participants revealed that long‐term noise exposure was associated with increased arterial inflammation and cardiovascular risk, potentially through enhanced amygdala metabolic activity and subsequent sympathetic activation [20].

The brainstem, which comprises the midbrain, pons, and medulla oblongata, contains key autonomic nuclei that govern cardiovascular function. The upper brainstem primarily coordinates adaptive responses to external stimuli. It integrates forebrain inputs related to nociception, emotion, and cognition and translates them into cardiovascular responses. In contrast, the lower brainstem acts as the central hub for regulating internal homeostasis, including blood circulation and respiration.

The periaqueductal gray modulates both pain perception and cardiovascular function through the ANS [21]. For instance, deep brain stimulation of the ventral periaqueductal gray alters heart rate variability (HRV), by increasing parasympathetic tone while reducing pain perception [21]. Furthermore, aversion‐related stimuli increase activity and functional connectivity within the amygdala–periaqueductal gray pathway, eliciting parasympathetically mediated bradycardia [22].

The pontine locus coeruleus, the primary noradrenergic nucleus in the mammalian central nervous system (CNS), may influence susceptibility to sudden unexpected death in epilepsy (SUDEP) via cardiac β1‐adrenergic receptors. This finding identifies a novel heart–brain interaction pathway contributing to seizure‐related apnea and fatal outcomes [23]. Separately, a randomized controlled trial demonstrated that daily slow‐paced breathing in older adults increases HR oscillations and hippocampal volume, with this effect depending on locus coeruleus noradrenergic input, suggesting neuroprotective potential [24]. The lateral parabrachial nucleus modulates the cardiovascular chemoreflex. In a rat model, microinjection studies revealed that blocking γ‐aminobutyric acid type A (GABA_A) receptors enhanced the chemoreflex‐mediated pressor response but reduced bradycardia, whereas blocking α_2_‐adrenergic receptors selectively inhibited the pressor response. These results indicate that GABAergic inhibition and adrenergic excitation in this nucleus independently regulate distinct cardiovascular components of the chemoreflex without significant crosstalk [25].

Multiple nuclei within the medulla oblongata are critical for heart–brain interactions. The ventrally located vasomotor center regulates vascular tone, BP, and blood flow by governing sympathetic outflow. The ventrolateral medulla comprises two functionally distinct subdivisions: the RVLM and CVLM. The RVLM is the primary excitatory center for sympathetic activity [26], while the CVLM is a key relay in the baroreceptor reflex and inhibits sympathetic outflow originating from the RVLM. In a chronic heart failure (CHF) rat model, glutamate receptor antagonism in the RVLM was shown to reduce basal BP, renal sympathetic nerve activity, and presympathetic neuronal discharge [27]. These findings indicate that an imbalance between excitatory and inhibitory inputs to the RVLM contributes to elevated sympathetic outflow in CHF.

The dorsal medullary vagal complex comprises the NTS, nucleus ambiguus, dorsal motor nucleus of the vagus, and area postrema. The NTS serves as the primary integrator of chemosensory inputs, receiving signals from peripheral chemoreceptors in the carotid and aortic bodies [28], as well as from central chemoreceptors within the medulla [29]. It also processes mechanosensory signals from cardiac chambers, blood vessels, and visceral walls [30]. Following integration, the NTS relays this information to key brainstem centers, including the medullary pre‐Bötzinger complex and the pontine locus coeruleus and parabrachial nucleus, to coordinate respiration and cardiovascular dynamics [31]. Studies indicate that brain‐derived neurotrophic factor (BDNF) in the dorsomedial NTS provides tonic inhibition of sympathetic activity via its receptor, tropomyosin receptor kinase B (TrkB), which is crucial for normal baroreflex function. In CHF, disruption of this BDNF–TrkB pathway contributes to sympathetic overactivation and baroreflex impairment [32]. Furthermore, in spontaneously hypertensive rats, dysregulated acid‐sensing ion channel (ASIC) 1 signaling in the NTS contributes to the exaggerated cardiopulmonary response to hypercapnic challenge [33].

The nucleus ambiguus is the principal vagal nucleus regulating cardiac and pulmonary functions [34]. Notably, renin is expressed specifically within its cholinergic neurons. A cholinergic neuron‐specific Ren‐a knockout mouse model confirmed that this locally produced renin contributes to autonomic balance and cardiovascular stability [35]. Direct carbon‐fiber microelectrode recordings from preganglionic neurons in the nucleus ambiguus and dorsal motor nucleus of the vagus demonstrated that acute exercise potently activates, rather than suppresses, central vagal drive. Furthermore, chronic exercise training increased both the basal firing rate and activity‐evoked responses of these neurons, providing direct neurophysiological evidence that exercise enhances vagal tone [36]. Research has shown that the cardiovascular baroreflex shares a neural substrate with sleep regulation. Specifically, activating pressure‐sensitive neurons in the NTS and their downstream pathways, including the GABAergic pathway in the CVLM and the cholinergic cardiac pathway in the nucleus ambiguus, simultaneously promotes nonrapid eye movement sleep while reducing BP and HR. This finding provides insights into shared neural circuits that may explain the frequent comorbidity of CVDs and sleep disorders [37].

#### 2.1.2 The Extrinsic Cardiac Nervous System: A Relay Station for Heart–Brain Signaling

The extrinsic cardiac nervous system (ECNS) comprises sympathetic and parasympathetic efferent pathways that convey signals from the CAN to the heart. It relays central autonomic output to the cardiac conduction system and myocardium [38].

##### 2.1.2.1 Parasympathetic Outflow

Preganglionic parasympathetic neurons reside in the medullary nucleus ambiguus and dorsal motor nucleus of the vagus. Their cholinergic fibers project through the vagus nerves to cardiac ganglia, where they converge with sympathetic nerves within the cardiac plexus [9, 39]. These parasympathetic nerves primarily innervate key cardiac structures, including the sinoatrial node, atria, atrioventricular node, and ventricular conduction system. Using retrograde tracing, single‐cell sequencing, and optogenetics, Krasnow and colleagues identified a dual‐circuit architecture for cardiac parasympathetic regulation in mice [34]. They identified two molecularly, anatomically, and functionally distinct subtypes of cardiac‐projecting neurons in the nucleus ambiguus: the “ambiguus cardiovascular” neurons, which form a dedicated cardiac pathway, and the “ambiguus cardiopulmonary” neurons, which project to both cardiac and pulmonary ganglia [34]. These findings suggest that cardiac parasympathetic control is mediated by two parallel pathways: a dedicated cardiovascular circuit and a coordinated cardiopulmonary circuit [34].

##### 2.1.2.2 Sympathetic Outflow

Sympathetic preganglionic neurons originate from the intermediolateral cell column of the thoracolumbar spinal cord, and their activity is driven primarily by inputs from the RVLM [9]. Their preganglionic axons synapse with postganglionic neurons within the paravertebral sympathetic chain ganglia. Over 90% of postganglionic neuronal somata are located in the middle cervical (C3–C6) and cervicothoracic (stellate) ganglia (C5–T3), with the remainder distributed among the superior cervical (C1–C3), vertebral (C4–C7), and thoracic ganglia [5]. The postganglionic sympathetic fibers then intermingle with parasympathetic fibers to form the cardiac plexus [9, 39]. These sympathetic nerve endings release norepinephrine (NE) and predominantly innervate the atrial myocardium, with less extensive projections to the ventricles [38].

Autonomic efferent pathways modulate HR dynamics by transmitting sympathetic and parasympathetic signals to the sinoatrial node. Consequently, the spectral analysis of HRV is widely used to assess cardiac autonomic modulation. In the HRV power spectrum, the low‐frequency component, ranging from 0.04 to 0.15 Hz, reflects mixed autonomic influences, including both sympathetic and parasympathetic activity. In contrast, the high‐frequency component, ranging from 0.15 to 0.40 Hz, primarily reflects parasympathetic activity, but this interpretation is valid only when the respiratory rate falls within the 0.15–0.40 Hz range [40]. Thus, HRV is an important biomarker of autonomic function and can be used to assess dynamic changes in heart–brain interactions.

#### 2.1.3 The ICNS: The Heart's “Little Brain”

The ICNS is composed of more than 500 ganglionated plexuses situated within the epicardial fat pads and myocardial wall [9]. This network encompasses over 14,000 neurons, including afferent neurons, efferent neurons, and interneurons, which form intricate local reflex circuits [9, 41]. These plexuses are concentrated in five primary regions: the superior and posterior right atrium, superior left atrium, the roots of the great vessels, the atrioventricular groove, and the interatrial septum [9]. Through its highly interconnected ganglia, the ICNS processes sympathetic and parasympathetic inputs from the ECNS and directly regulate cardiac electrophysiology and contractility. Owing to its capacity to initiate and fine‐tune cardiac rhythm, the ICNS is often referred to as the heart's “little brain” [42].

Research is increasingly clarifying how distinct ganglionated plexuses exert region‐specific control over cardiac function. For instance, ganglia at the aortic root mediate stress‐induced coronary vasoconstriction through connections with the pericoronary neural plexus [43]. Similarly, the right atrial ganglionated plexus contributes to vagal modulation of sinoatrial node activity [44, 45]. These findings provide a rationale for developing ICNS‐targeted neuromodulatory and ablative therapies. Consistent with this, a 3‐year follow‐up study showed that botulinum neurotoxin injection into the ICNS reduces arrhythmia recurrence [46]. However, in the AFACT trial, neural ablation did not improve freedom from arrhythmia recurrence in patients with atrial fibrillation (AF) and was associated with an increased need for permanent pacemaker implantation [47]. A recent multidisciplinary study combining neuronal electrophysiology, optical mapping, immunohistochemistry, tissue clearing, and right atrial plexus ablation constructed an integrated neuroanatomical map of the right atrial ganglionated plexus–sinoatrial node complex in porcine and human hearts. This research confirmed that the right atrial ganglionated plexus modulates sinoatrial node automaticity, atrioventricular conduction, and left ventricular contractility, demonstrating a direct role for intrinsic cardiac neurons in pacemaker control [48]. Future studies should delineate the functional properties of other ICNS components in the mammalian heart, thereby guiding the development of targeted therapeutic frameworks.

### 2.2 Biochemical Pathways

The biochemical pathway comprises diverse endogenous bioactive molecules, including hormones, NPY, and mediators of acute and chronic inflammation, that collectively regulate cerebral and cardiovascular functions and autonomic tone.

#### 2.2.1 Natriuretic Hormones

The natriuretic hormone system primarily includes natriuretic peptides, gastrointestinal peptides, and endogenous cardiotonic steroids [49]. Natriuretic peptides are classified into three types: atrial natriuretic peptide, B‐type natriuretic peptide (BNP), and C‐type natriuretic peptide. Atrial natriuretic peptide and BNP are primarily secreted by the atria, whereas C‐type natriuretic peptide is produced by vascular endothelial cells. The primary physiological role of the natriuretic peptide system is to counterbalance the renin–angiotensin–aldosterone system by promoting natriuresis, diuresis, vasodilation, and by inhibiting myocardial remodeling, thereby reducing BP and cardiac workload [50]. Notably, natriuretic peptides and their receptors are widely expressed in the mammalian CNS. Consequently, beyond their renal and cardiovascular effects, these peptides modulate diverse cerebral processes [49, 51, 52]. Evidence suggests their involvement in regulating neurodevelopment, synaptic transmission, blood–brain barrier (BBB) integrity, cerebral fluid homeostasis, neuroinflammation, and neuroprotection [51]. Studies indicate that exogenous natriuretic peptides may have therapeutic potential not only for Parkinson's disease but also for other neurodegenerative disorders associated with dysregulated Wnt signaling [53].

Endogenous cardiac steroids are a group of compounds synthesized primarily in the adrenal glands and hypothalamus. They share structural and functional similarities with exogenous cardiotonic glycosides, such as digoxin, by binding to and inhibiting Na^+^/K^+^‐ATPase, a mechanism long exploited in HF management [49]. Functionally, they can be considered endogenous counterparts of digoxin. As Na^+^/K^+^‐ATPase is an essential enzyme expressed ubiquitously, these steroids exert pleiotropic effects on diverse cell types, including cardiomyocytes and neurons. In the heart, they inhibit cardiomyocyte sarcolemmal Na^+^/K^+^‐ATPase, elevating intracellular Na^+^. This rise in Na^+^ promotes Ca^2^^+^ influx via the reverse‐mode Na^+^/Ca^2^^+^ exchanger, thereby increasing cytosolic Ca^2^^+^ concentration and resulting in positive inotropy and enhanced contractility. However, under pathological conditions, persistently elevated cardiac steroid levels can induce cardiomyocyte calcium overload and promote fibrosis, thereby accelerating cardiac dysfunction [54]. In the brain, endogenous cardiac steroids of hypothalamic origin target various Na^+^/K^+^‐ATPase isoforms, contributing to sodium‐sensitive hypertension via activation of the central renin–angiotensin system [49]. Thus, dysregulated cardiac steroid levels in the brain and impaired Na^+^/K^+^‐ATPase function represent key shared mechanisms linking cardiovascular and psychiatric disorders [49, 54].

#### 2.2.2 Neuropeptide Y

NPY is a highly conserved neuropeptide abundantly distributed throughout the central and peripheral nervous systems. It is coreleased with NE from postganglionic sympathetic nerve terminals, where it acts as a cotransmitter [55]. Unlike classical neurotransmitters, NPY exhibits a substantially longer half‐life and more sustained biological effects [55]. As the most abundant neuropeptide in the heart, NPY modulates various cardiovascular functions. Its plasma levels rise sharply under conditions of sympathetic hyperactivity, including stress, ischemia, and HF, underscoring NPY's pathophysiological significance in CVDs [55].

NPY holds considerable prognostic value. In patients with acute MI, elevated plasma NPY levels are associated with microvascular dysfunction, larger infarct size, and long‐term adverse outcomes, supporting their potential use as a clinical biomarker for assessing myocardial injury [56]. Among HF patients receiving cardiac resynchronization therapy, a coronary sinus NPY concentration of ≥130 pg/mL is associated with a 9.5‐fold higher mortality risk, likely reflecting increased sympathetic drive [57]. Axonal modulation therapy, which targets the paravertebral sympathetic chain, produces a region‐specific reduction in sympathetically mediated NPY release. This finding provides a conceptual basis for developing closed‐loop antiarrhythmic systems targeting sympathetic cotransmission [58].

Lovelace and colleagues identified the molecular and circuit basis of the Bezold–Jarisch reflex. They discovered that vagal sensory neurons expressing NPY Y2 receptors constitute the cellular substrate of a direct neural circuit from the cardiac ventricles to the area postrema in the brainstem. Optogenetic stimulation of this pathway elicited characteristic cardiovascular responses, including bradycardia, hypotension, and reduced cerebral perfusion—and directly triggered syncope accompanied by widespread suppression of neuronal activity across the brain [59]. Crucially, syncope still occurred, albeit with delayed onset, even when bradycardia and hypotension were pharmacologically blocked by atropine. This finding reveals a direct neural circuit mechanism for syncope that operates independently of classical hemodynamic changes [59].

Furthermore, NPY mediates bidirectional heart–brain interactions, facilitating signaling from the brain to the heart and from the heart to the brain. A study in a porcine model demonstrated that cardiac pressure overload and ovariectomy‐induced sex hormone deficiency synergistically impaired cerebrovascular function and spatial memory. The underlying mechanism involves enhanced vasoconstrictive responses of pial arteries to NPY, suggesting that NPY contributes to the development of cardiac dementia in HF [60].

#### 2.2.3 Inflammatory Mediators

Beyond inducing a local inflammatory response, cardiac injury can trigger remote neuroinflammation. A seminal study identified a “heart–brain–immune” axis following MI: cardiac damage promotes monocyte infiltration into the brain, promoting protective sleep that subsequently limits excessive cardiac inflammation driven by sympathetic activation. This finding highlights the critical protective role of sleep in postinfarction recovery and identifies immune‐neural interactions as the underlying mechanism [8]. Another study elucidated a neural pathway in which cardiac pressure overload is sensed by afferent nerves, driving ATP release from sympathetic efferents. This extracellular ATP stimulates the purinergic P2×7 receptor, thereby activating the NLRP3 inflammasome in cardiac nonimmune cells to produce IL‐1β and drive adaptive hypertrophy [61]. This mechanism suggests a novel pathway through which heart–brain interactions may regulate cardiac inflammation and hypertrophy via neural signaling [61]. Systemic imaging of the 18‐kDa translocator protein (TSPO) imaging revealed that acute MI triggers a biphasic heart–brain inflammatory response: an initial phase of concurrent myocardial and neuroinflammation, followed by a later stage of CHF characterized by TSPO upregulation associated with impaired cardiomyocyte mitochondrial function and recurrent neuroinflammation [62]. Targeting this inflammatory axis may thus represent a therapeutic strategy to improve both cardiac and neurological outcomes.

The proposed “stroke‐induced heart injury” framework posits that poststroke dysregulation of the CAN triggers sympathetic overactivity and a systemic inflammatory response, thereby contributing to the clinical manifestations of “stroke–heart syndrome,” including myocardial injury, arrhythmias, and HF [5]. Subsequent research identified a critical HDAC6–SIRT1–NLRP3 inflammatory axis in the poststroke brain that promotes cardiac damage by driving systemic inflammation and sympathetic activation. Concurrently, a brain‐targeted nanoplatform (DATS/MION‐LPE) was developed to precisely modulate this pathway, supporting the suppression of neuroinflammation as a viable strategy for preventing poststroke cardiac complications [63]. Parallel research showed that acute brain ischemia can induce persistent proinflammatory memory phenotype in myeloid innate immune cells. This IL‐1β‐driven memory state, established via epigenetic reprogramming, promotes sustained monocyte and macrophage infiltration into distal organs such as the heart, leading to myocardial fibrosis and diastolic dysfunction. Notably, neutralizing IL‐1β or inhibiting monocyte recruitment with CCR2/5 antagonists effectively prevents the development of poststroke cardiac complications [64].

### 2.3 Mechanical Pathways

The mechanical pathway of heart–brain interactions mediates afferent communication from the heart to the brain and is primarily enabled by baroreceptors (mechanosensitive receptors) and cardiopulmonary dynamics.

Virtually all somatic cells sense and transduce mechanical forces into electrochemical or biochemical signals through mechanosensation, a process fundamental to cellular physiology, organogenesis, and systemic homeostasis [65]. PIEZO1 and PIEZO2 are widely expressed, nonselective mechanosensitive cation channels that detect mechanical stimuli, such as membrane tension, through their trimeric, propeller‐like structure [66].

PIEZO channels display a cell‐specific distribution pattern: PIEZO1 is primarily expressed in brain capillary endothelial cells and astrocytes, where it senses fluid shear stress; PIEZO2 is predominantly found in somatosensory neurons, with low expression in glial cells, and its expression level is negatively correlated with the extent of cellular deformation [3]. Within the cardiovascular system, PIEZO channels are pivotal for transducing hemodynamic forces, such as shear stress, into biological signals, thereby regulating endothelial function, vascular cell behavior, erythrocyte homeostasis, platelet aggregation, and arterial pressure [66]. As core mediators of cardiac mechanotransduction, PIEZO channels are integral to cardiac valve and coronary artery development, baroreceptor reflex modulation, and homeostatic maintenance in the adult heart. Thus, they provide a coherent mechanism for the continuous mechanical control of cardiac architecture and function from embryogenesis through adulthood [67]. Furthermore, PIEZO channels contribute to pathological cardiovascular remodeling. Their dysregulation promotes key pathological processes, including baroreflex impairment, pathological cardiac hypertrophy, fibrosis, ventricular dysfunction, and inflammation, thereby supporting their potential as targets for precision therapy [67].

Studies have identified a novel, rapid pathway for heart–brain interactions, demonstrating that brain neurons can directly sense cardiovascular pressure pulsations. In rodents, mitral cells of the olfactory bulb express mechanosensitive ion channels such as PIEZO2, enabling direct conversion of arterial pulsations into electrical signals and entraining neuronal firing to the cardiac rhythm within ∼20 ms. This nonsynaptic transduction mechanism appears to operate broadly across multiple brain regions. Based on these findings, researchers proposed the “heartbeat sentinel neuron” hypothesis, which posits that such neurons form a whole‐brain network through which cardiac interoception directly regulates brain functions, including arousal, cognition, and emotion [68].

The baroreflex regulates cardiac function by detecting arterial pressure oscillations. Each heartbeat generates a pressure pulse that radially stretches elastic vessel walls, activating mechanosensory endings that signal the CNS about vascular stretch, pulse frequency, and mean arterial pressure. Through central autonomic circuits, the baroreflex reduces sympathetic outflow and increases parasympathetic activity, thereby lowering HR, cardiac output, and peripheral vascular resistance [66].

PIEZO1 and PIEZO2 are key molecular sensors in baroreceptors that transduce arterial wall distension [66]. Quantitative immunohistochemistry revealed widespread expression of both channels in sensory (petrosal) and sympathetic (superior cervical) ganglia, with PIEZO2 detected in >85% of neurons. In the carotid body, PIEZO2 shows broad expression in Type I glomus cells and nerve fibers, while PIEZO1 localization is more restricted; notably, both channels exhibit high coexpression in Type II glomus cells [69]. Genetic studies confirmed the essential role of PIEZO channels. Conditional double knockout of *Piezo1*/*Piezo2* in mouse nodose‐petrosal ganglion neurons abolished both reflex bradycardia in response to pharmacological challenges and electrical responses in the aortic depressor nerve. In vivo, double‐knockout mice exhibited labile hypertension, exercise‐induced hypertension, and markedly increased BP variability, recapitulating key features of baroreflex failure [70]. Despite functional overlap, PIEZO2 plays a dominant role. PIEZO2‐expressing neurons in vagal and petrosal nerves form a unique “claw‐like” structure around the aortic arch, extending delicate “reticulated endings” that sense vessel dilation [71]. Conditional ablation of these neurons abolished baroreflex regulation of HR and BP, while optogenetic activation induced reflex hypotension and bradycardia [71]. In rat models, PIEZO2 function is specifically suppressed by the E3 ubiquitin ligase Nedd4‐2 during hypertension, impairing baroreflex sensitivity and increasing sympathetic tone, thereby suggesting a potential molecular mechanism underlying hypertension [72]. Recent work showed that *Piezo2* knockout or ablation of PIEZO2‐positive neurons in the vagal ganglia abolished heartbeat‐synchronized vagal activity. These manipulations also caused orthostatic hypotension and impaired cardiovascular stability during blood loss. These findings suggest that PIEZO2‐positive vagal afferent neurons monitor cardiac mechanical loading and changes in blood volume, thereby initiating reflex pathways that maintain circulatory homeostasis [73].

Notably, individual knockout of either *Piezo1* or *Piezo2* has minimal impact on baroreflex function, whereas dual knockout severely disrupts baroreflex function, indicating that baroreception is not mediated by a single mechanosensitive molecule [70, 74]. Consistent with this, PIEZO2 expression is compensatorily upregulated following PIEZO1 downregulation, revealing a cooperative and complementary relationship between these channels in baroreflex circuitry [75]. Furthermore, beyond PIEZO channels, other mechanosensitive channels, including transient receptor potential (TRP), epithelial sodium channel, and ASIC families, also contribute to baroreflex mechanotransduction [74].

Finally, respiratory activity couples with cardiac rhythm through both mechanical and neural pathways. The mechanical pathway modulates cardiac chamber filling and aortic pressure, while the neural pathway uses the NTS as a central integration hub. Respiration‐derived mechanical signals are detected by specialized receptors and relayed to the CAN, enabling fine‐tuned control of cardiovascular function [3].

## 3 Heart–Brain Interactions in Diseases

Dysregulation of heart–brain interactions contributes to the pathogenesis and progression of diverse cardiovascular and neurological disorders. Building on the physiological foundations outlined above, this section examines TTS, epileptic heart, SHS, HF, cardiac dementia, and psychocardiology as representative conditions to elucidate the pathophysiological mechanisms and clinical implications of heart–brain interactions.

### 3.1 Takotsubo Syndrome

TTS, or stress‐induced cardiomyopathy, is characterized by acute, reversible left ventricular dysfunction and accounts for approximately 1–4% of patients initially suspected of having acute coronary syndrome (ACS) [76]. It predominantly affects postmenopausal women and is frequently triggered by major physical or emotional stress, leading to its colloquial name, “broken heart syndrome” [76]. Recent studies have revealed that positive life events can also precipitate a subtype of TTS known as “happy heart syndrome” [77]. Data from the International Takotsubo Registry indicate that among 485 TTS patients with clearly identified emotional triggers, 4.1% of cases were precipitated by positive events. These patients exhibited clinical presentations, electrocardiographic parameters, laboratory findings, and 1‐year outcomes comparable to those with negative emotional triggers. However, subgroup analysis revealed a significantly higher prevalence of mid‐ventricular ballooning in the “happy heart” group than in the “broken heart” group (35.0 vs. 16.3%). This suggests that despite opposing emotional valence, both positive and negative events may trigger TTS through shared neural and emotional pathways [78].

The strong association between physical or emotional stressors and TTS episodes implicates the CNS as a key contributor to the pathogenesis of TTS. Multicenter studies report that 55.8% of patients with TTS have comorbid psychiatric or neurological conditions [79]. Furthermore, both neurological disorders (HR = 1.76) and psychiatric disorders (HR = 1.77) are independent predictors of TTS recurrence [80].

#### 3.1.1 Autonomic Dysfunction

Catecholamine‐induced myocardial injury is the most substantiated pathophysiological mechanism of TTS [81]. Although it does not fully explain all features of TTS, it supports the presence of autonomic dysfunction characterized by sympathetic hyperactivity. HRV analysis reveals significantly reduced indices during the acute phase, with gradual recovery in the subacute phase and near‐normalization by 3 months [82]. This dynamic pattern indicates sympathetic activation with concomitant parasympathetic inhibition in the acute phase, followed by gradual recovery of autonomic regulation. Microneurography confirms sympathetic overactivity in patients with TTS, accompanied by reduced spontaneous baroreflex sensitivity [83]. Furthermore, ^1^ ^2^ ^3^I‐metaiodobenzylguanidine scintigraphy demonstrates a reduced heart/mediastinum ratio and increased washout rate in patients with TTS. Single‐photon emission computed tomography imaging shows only minor perfusion changes, suggesting functional impairment of central sympathetic neurotransmission [84].

Neuroimaging studies provide direct evidence of CAN dysfunction in TTS. Single‐photon emission computed tomography imaging reveals increased cerebral blood flow in the hippocampus, brainstem, and basal ganglia, alongside decreased perfusion in the prefrontal cortex during the acute TTS phase; these changes normalize during recovery [85]. MRI studies demonstrate reduced volumes in frontal lobe regions and CAN structures in acute TTS [86]. Furthermore, patients with TTS exhibit widespread functional connectivity abnormalities across 384 brain regions, particularly involving connectivity among the right anterior insula, temporal lobe, and precuneus [86].

Notably, the right insula, a key regulator of sympathetic tones, shows both volume loss and reduced functional connectivity in TTS. As a CAN hub, the insula integrates sensory, interoceptive, emotional, and autonomic information, thereby mediating cardiac interoception and cardiovascular responses to emotional stimuli [87]. Clinically, insular damage is associated with QT prolongation, myocardial injury, elevated catecholamines, and TTS occurrence [88]. However, another MRI study reported conflicting findings: increased left insular volume, with enhanced structural connectivity but reduced functional connectivity [89]. The reasons for these discrepancies remain unresolved.

Resting‐state functional MRI (fMRI) studies demonstrate reduced functional connectivity in both parasympathetic and sympathetic subnetworks, including key nodes such as the amygdala, hippocampus, and insula [90]. Similar reductions are observed in the default mode network and distributed limbic regions [90]. Multimodal analyses reveal distinct brain remodeling in acute TTS: reduced cortical surface area with increased thickness, hippocampal shrinkage, and thalamic and insular enlargement. A structure–function dissociation is evident: despite enhanced structural connectivity, functional connectivity in emotion and autonomic networks is reduced [89]. Collectively, these findings suggest that impaired autonomic–limbic integration contributes to the pathophysiology of TTS.

The insula may constitute a key neuroanatomical substrate underlying the sex differences observed in TTS [91]. Functionally, the right insula is predominantly linked to sympathetic outflow, while the left insula is more involved in parasympathetic modulation. Postmenopausal estrogen depletion may reduce left hemispheric activity, thereby favoring sympathetic dominance [91]. This mechanism likely explains the marked female predominance, with women accounting for approximately 90% of TTS cases [92]. Additionally, age‐related increases in sympathetic drive, combined with declining vagal tone and impaired baroreflex sensitivity, collectively increase myocardial sensitivity to catecholamines. This pathophysiological shift may underlie the characteristic age‐specific distribution of TTS onset [79].

#### 3.1.2 Emotional Abnormalities

TTS can be triggered by both severe physical and psychological stressors [81]. It may also be triggered by various cardiac and noncardiac conditions, including neurological disorders such as intracranial hemorrhage and epilepsy, pheochromocytoma, and other acute critical illnesses [81]. Similarly, the substantial physical and psychological burden associated with malignancy can trigger TTS [93].

Epidemiological data indicate that approximately one‐third of patients with TTS have comorbid psychiatric conditions, particularly depression and anxiety disorders [81]. Notably, the prevalence of comorbid psychiatric or neurological disorders is sevenfold higher in patients with TTS than in those with ACS. A majority also exhibit a Type D personality, characterized by negative affectivity and social inhibition [94]. This association may stem from exaggerated cardiovascular reactivity to stress and the characteristic emotional inhibition in Type D individuals, which may potentiate catecholamine release. This psychologically mediated “hyper‐responsive” stress pattern directly affects the heart through neuroendocrine pathways, thereby increasing susceptibility to TTS [95]. Integrated neuroimaging and psychometric assessments reveal elevated anxiety and precuneus hyperconnectivity in patients with TTS, suggesting a predisposition to negative emotional experiences and impaired emotion regulation [96]. Consequently, patients with TTS exhibit significant impairments in emotion regulation.

As previously noted, the limbic system plays a primary role in mediating emotion‐related cardiovascular responses. Neuroimaging studies have highlighted the involvement of the amygdala and the broader limbic network in TTS pathophysiology. MRI studies reveal structural alterations in the limbic circuitry of patients with TTS, including cortical thinning in the anteroventral insula, reduced amygdala gray matter volume, and diminished structural connectivity [97]. Resting‐state fMRI shows heightened functional integration among brain regions involved in interoceptive pain perception and negative affective processing [98]. ^1^ ^8^F‐fluorodeoxyglucose positron emission tomography/computed tomography (^1^ ^8^F‐FDG PET/CT) demonstrates that elevated baseline amygdala activity predicts subsequent TTS risk (HR = 1.643) and correlates with earlier disease onset [99]. These findings indicate that amygdalar hyperactivity is associated with an increased risk of TTS, potentially by amplifying neurohormonal and physiological responses to stress [100]. Notably, this aberrant neurobiological signature may precede the clinical onset of TTS by several years [99]. Cerebral metabolic imaging further supports chronic limbic hypermetabolism in TTS. ^1^ ^8^F‐FDG PET/CT shows persistently increased glucose metabolism in the amygdala, hippocampus, and midbrain beyond the acute phase [101]. This is accompanied by impaired prefrontal regulation (an elevated amygdala‐to‐prefrontal activity ratio), suggesting that dysregulated emotional circuitry underlies TTS at the neurobiological level [101]. Consistent with this, chronic mild stress appears more important in TTS pathogenesis than previously recognized. It dysregulates the HPA axis and ANS via reduced glucocorticoid and β‐adrenergic receptor sensitivity, leading to prolonged immune suppression and chronic inflammation [102]. Therefore, interventions targeting stress‐related neural activity represent a promising strategy for TTS prevention.

Together, these studies reveal substantial alterations in emotional processing regions in patients with TTS. However, apart from the prospective association between baseline amygdala activity and subsequent TTS risk reported by Tawakol et al. [99], most evidence derives from cross‐sectional studies. Thus, it remains unclear whether these cerebral alterations represent causes or consequences of TTS, a distinction that requires clarification in future prospective studies.

Emotional disturbances trigger TTS through integrated neurobiological mechanisms. As discussed above, psychological disturbances are associated with CNS dysfunction and ANS dysregulation. Chronic psychiatric conditions, including depression, anxiety, and posttraumatic stress disorder, are associated with limbic system alterations, such as those involving the amygdala and hippocampus, that impair autonomic regulation, promoting sympathetic overactivation and relative parasympathetic suppression during stress. Psychoneuroendocrine pathways further mediate this connection. Chronic stress or psychiatric disorders disrupt HPA axis regulation, manifesting as a blunted cortisol stress response. This blunted response may reduce restraint on catecholamine release, promoting sympathoadrenal medullary axis overactivation during acute stress. The resulting “catecholamine storm,” combined with autonomic dysfunction and low‐grade inflammation, collectively affect the myocardium and may trigger TTS [95].

#### 3.1.3 Integrated Heart–Brain Management of TTS

TTS carries an in‐hospital mortality rate comparable to that of acute ST‐segment elevation MI, and approximately one‐eighth of patients experiencing recurrence within 5 years. Long‐term follow‐up reveals an all‐cause mortality rate of 5.6% per patient‐year and a rate of major adverse cardiac and cerebrovascular events rate of 9.9% per patient‐year [79], highlighting the substantial long‐term clinical burden of TTS.

According to the international multicenter GEIST registry, 17.0% of patients with TTS had comorbid neurological diseases, most commonly cerebrovascular events (39.0%), neurodegenerative diseases (30.7%), migraine (10.0%), epilepsy (9.5%), and brain tumors (5.0%). Neurological comorbidities were associated with prolonged hospitalization, increased in‐hospital complications (27.0 vs. 16.0%), and higher mortality (6.5 vs. 2.8%). Multivariate analysis identified neurological diseases as independent predictors of both 60‐day (OR = 1.78) and long‐term mortality (HR = 1.72). Patients with neurodegenerative diseases had the worst prognosis, whereas those with migraine had relatively favorable outcomes. These findings underscore the need for early identification and risk stratification of neurological comorbidities in patients with TTS [103].

The InterTAK diagnostic criteria incorporate emotional triggers, neurological diseases, and psychiatric disorders into the diagnostic assessment, thereby improving TTS diagnostic specificity [104, 105]. In an independent validation cohort, the InterTAK scoring system achieved an area under the curve (AUC) of 0.901. At a cutoff of 40 points, it demonstrated 89% sensitivity and 91% specificity for TTS diagnosis, indicating excellent discriminative ability [105]. Integrated multimodal brain MRI and machine learning analysis revealed consistent structural and functional alterations in the brain's emotional–autonomic regulatory system in patients with TTS, with a discrimination accuracy exceeding 82% [106]. Current methods for assessing sympathetic activity, including HRV, BP variability, and intermittent catecholamine testing, have inherent limitations that hinder continuous and precise monitoring. The emerging technique of skin nerve activity recording provides a noninvasive surrogate marker of sympathetic output from the cardiac stellate ganglion, offering a new approach for dynamic sympathetic assessment in TTS and HF [107].

The TTS management algorithm proposed by Ghadri et al. offers a structured framework for clinical management [108]. Cognitive behavioral therapy (CBT) reduces amygdala activity [109], suggesting its potential relevance for TTS prevention [99]. A randomized controlled trial demonstrated that a 12‐week program of exercise training or CBT promoted neurobiological recovery in patients with TTS. Both interventions increased cortical volume, decreased insular volume, and reduced the cortisol stress response; CBT additionally improved inflammatory markers and subjective stress scores [110]. Notably, these improvements occurred independently of the spontaneous recovery of cardiac function, indicating that behavioral interventions may accelerate recovery by modulating the limbic–autonomic axis [110].

**FIGURE 1:**
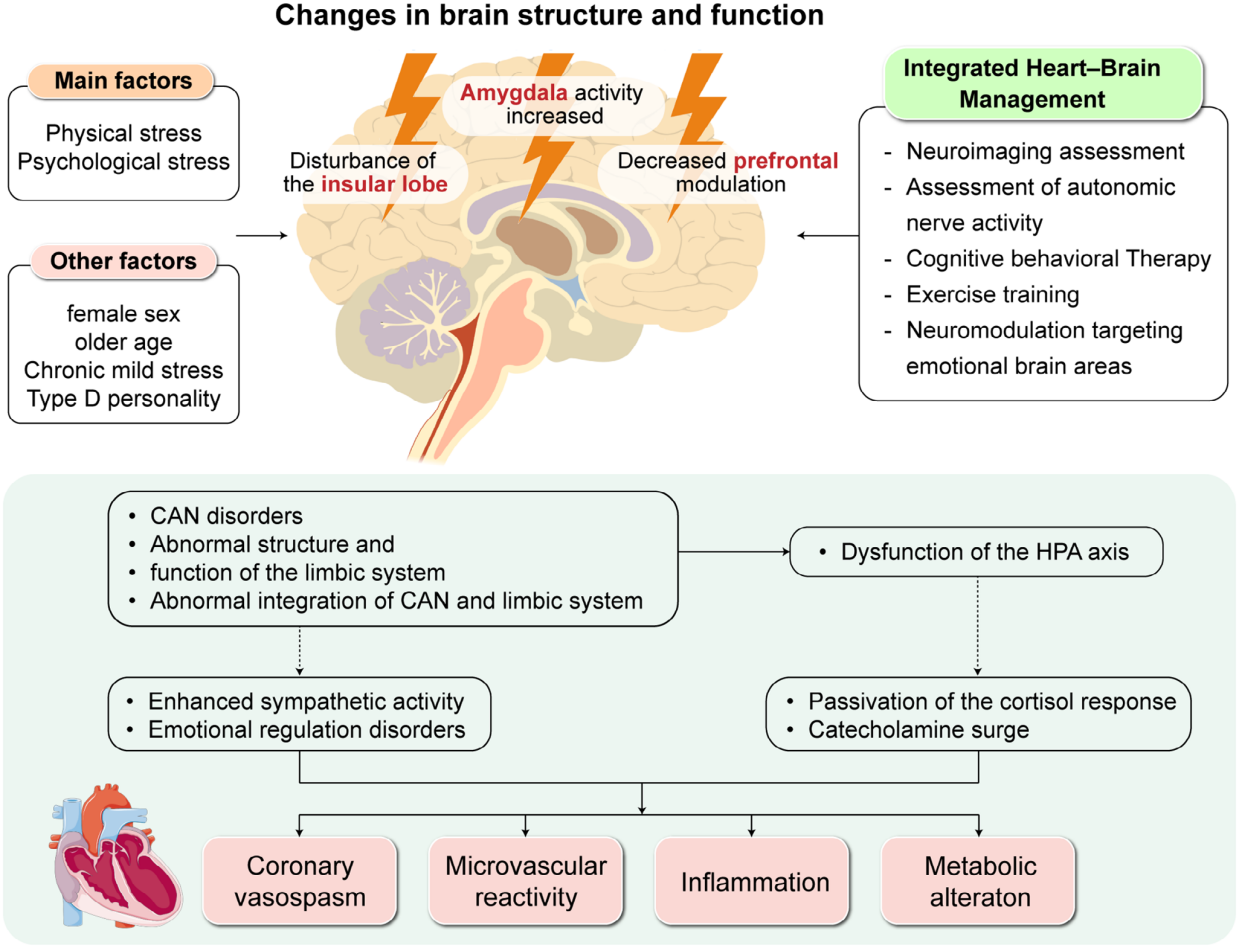
Heart–brain interactions in Takotsubo syndrome. Physical and psychological stressors, together with predisposing factors, may disrupt brain regions involved in autonomic and emotional regulation, including the insular lobe, amygdala, and prefrontal cortex. Dysfunction within the central autonomic network, limbic system, and HPA axis may promote sympathetic overactivation, catecholamine release, and impaired emotional regulation, thereby contributing to coronary vasospasm, microvascular dysfunction, inflammation, and metabolic alterations. Integrated heart–brain management may include neuroimaging assessment, autonomic activity monitoring, cognitive behavioral therapy, exercise training, and targeted neuromodulation. CAN, central autonomic network; HPA, hypothalamic–pituitary–adrenal axis.

In summary, TTS represents a paradigm of dysregulated heart–brain interactions (Figure 1). Dysregulated interplay among the limbic system, the CAN, and the peripheral neuroendocrine axis underlies neurophysiological, perceptual, and hormonal abnormalities that culminate in myocardial stunning. This “emotion–brain–heart” interaction helps explain the term “broken heart syndrome.” Neuromodulation strategies targeting cerebral emotion integration centers, particularly the amygdala and insula, represent a promising therapeutic direction.

### 3.2 The Epileptic Heart

Epileptic seizures induce acute cardiac electrophysiological disturbances, including HR alterations, arrhythmias, cardiac arrest, and other electrocardiogram (ECG) abnormalities, and can even trigger TTS [111]. The underlying mechanism involves abnormal activation of autonomic brain regions during seizures, including the insula, orbitofrontal cortex, and temporal lobe. Through connections with brainstem autonomic centers, these regions dysregulate respiratory and cardiovascular function, causing ictal and postictal complications, including abnormal HR, BP fluctuations, and severe arrhythmias [112]. Chronic recurrent seizures cause structural cardiac damage through the epileptic heart process, characterized by myocardial fibrosis, accelerated atherosclerosis, ventricular dysfunction, and arrhythmias [6]. Consequently, epilepsy has distinct acute and chronic cardiac effects: acute ictal autonomically mediated tachyarrhythmias or bradyarrhythmias, and chronic repolarization abnormalities, such as QT prolongation and reduced HRV, which increase the risks of HF and arrhythmias. Complex heart–brain interactions underlie both SUDEP and sudden cardiac death [112].

A large‐scale cohort study confirmed an increased incidence of arrhythmias in patients with epilepsy (HR = 1.36). Polygenic risk score analysis indicated that this association is independent of genetic predisposition [113]. Notably, certain antiseizure medications, particularly carbamazepine (HR = 1.46) and valproate (HR = 1.55), are associated with an additional risk of arrhythmia. A potential mechanism involves their action on ion channels, such as sodium channels, expressed in both cardiac and neural tissues. Channel blockade or drug‐induced metabolic disturbances can provoke cardiac electrical instability [113, 114]. Conversely, cardiac disease is also associated with epilepsy development. A Danish nationwide cohort study showed that patients with congenital heart disease had a significantly increased epilepsy risk (RR = 2.3–3.7). Notably, even patients with congenital heart disease who had not undergone surgery had an elevated risk (RR = 3.1), suggesting that beyond surgical factors, congenital mechanisms, such as impaired fetal cerebral oxygen regulation, may increase epilepsy susceptibility through neurodevelopmental effects [115].

The following section examines the specific mechanisms underlying epilepsy‐induced arrhythmias and addresses strategies for integrated heart–brain management in patients with epilepsy.

#### 3.2.1 Autonomic Imbalance and Disruption of Cardiac Electrical Stability

Elevated sympathetic tone represents a key mechanism of cardiac dysfunction in patients with epilepsy [116, 117]. According to the classical view, abnormal ictal cortical discharges activate the sympathetic nervous system, triggering a catecholamine surge that disrupts cardiac electrical stability. The integrated epileptic heart model proposed by Verrier and colleagues further posits that recurrent catecholamine surges, hypoxemia, and adverse effects of antiseizure medications collectively cause both electrophysiological and mechanical cardiac dysfunction. This manifests as electrical instability, myocardial stunning, interstitial fibrosis, and accelerated atherosclerosis [6]. This hypothesis is supported by multimodal evidence. Stereo‐electroencephalography studies demonstrate a dynamic shift toward parasympathetic suppression and sympathetic activation during temporal lobe seizures, resulting in marked ictal tachycardia [118]. Echocardiographic and electrocardiographic analyses reveal that left ventricular stiffness in young patients with temporal lobe epilepsy correlates positively with the severity of autonomic dysfunction [119].

SUDEP constitutes a major cause of mortality in patients with epilepsy. The underlying mechanisms primarily involve CAN dysfunction and cardiopulmonary regulatory abnormalities. The pathophysiological process typically involves postictal autonomic imbalance, beginning with sympathetic hyperactivity (tachycardia, hypertension) that may shift toward vagal overactivity, causing central respiratory depression, bradycardia, and potentially culminating in cardiac arrest. Reduced HRV, reflecting impaired autonomic regulation, may serve as a potential biomarker for SUDEP [120].

#### 3.2.2 The Cardioneurological Channelopathy Theory

Ion channelopathies constitute a key pathophysiological link between cardiac and neurological disorders by simultaneously affecting both organ systems. Mutations in genes encoding voltage‐gated sodium, potassium, and calcium channels, as well as ryanodine receptor 2 (RyR2) receptor, can lead to the co‐occurrence of epilepsy syndromes, such as Dravet syndrome, and cardiac arrhythmias, such as Brugada syndrome and long QT syndrome. Beyond inherited abnormalities, the “second hit” hypothesis posits that epileptic seizures secondarily alter cardiac ion channel expression, establishing a persistent state of electrophysiological instability. This indicates that some forms of epilepsy may increase the risk of arrhythmias via channelopathies common to both the heart and brain [120].

The coexpression of multiple ion channels in the heart and brain explains why sodium channel‐targeting antiseizure medications can induce ECG abnormalities, including atrioventricular block and QT prolongation [121]. Mutations in genes encoding these ion channels underlie the co‐occurrence of epilepsy and arrhythmias, supporting the “cardio–cerebral channelopathy” concept. A seminal 2009 study revealed that KCNQ1 mutations, known to cause long QT syndrome Type 1, can also produce an epileptic phenotype. The encoded KvLQT1 channel is expressed not only in the heart but also in forebrain neuronal networks and brainstem nuclei. Its functional impairment disrupts neuronal repolarization, simultaneously triggering seizures and cardiac autonomic dysregulation [122]. This discovery supports a dual heart–brain pathogenic mechanism in channelopathies, providing new directions for the genetic diagnosis of epilepsy of unknown etiology and the prevention of sudden death. Major ion channels implicated in epilepsy–arrhythmia comorbidity include *KCNQ1*, *KCNQ2*, *KCNH2*, *KCNJ2*, *KCNA1*, *SCN1A*, *SCN2A*, *SCN5A*, *SCN8A*, *SCN10A*, *HCN1*, *HCN4*, *CACNA1C*, *CACNA2D1*, and *RYR2* [111].

Notably, epilepsy can induce secondary remodeling of cardiac ion channel expression, inducing electrophysiological abnormalities [123]. Animal studies confirm that epilepsy alters the expression of multiple cardiac ion channels, including voltage‐gated sodium channels (Nav1.1 and Nav1.5), potassium channels (Kv4.2 and Kv4.3), the sodium‐calcium exchanger NCX1, and hyperpolarization‐activated cyclic nucleotide‐gated channels (HCN2 and HCN4) [123]. The underlying pathophysiology involves autonomic dysfunction and myocardial remodeling. Chronic epilepsy increases cardiac adrenergic signaling, damages myocardial tissue, and promotes remodeling through abnormal autonomic output. Downstream effector molecules and transcription factors may further modulate cardiac ion channel expression [123]. In epileptic rat models, myocardial expression of Kv4.2 and Kv4.3 potassium channels is reduced, accompanied by β_1_‐adrenergic receptor downregulation and α_1_A‐adrenergic receptor upregulation [124]. This ion channel remodeling, combined with sympathetic hyperactivity, constitutes a key arrhythmogenic mechanism. Pharmacological studies demonstrate that beta‐blockers attenuate QTc prolongation and suppress ventricular tachycardia in epilepsy models, suggesting autonomic modulation as a potential therapeutic strategy [124]. Future clinical studies should determine whether cardiac ion channel remodeling occurs in patients with epilepsy, as it may underlie their proarrhythmic state.

#### 3.2.3 Integrated Heart–Brain Management of Epileptic Heart

Given these mechanisms, patients with epilepsy require systematic cardiac rhythm monitoring and management to mitigate long‐term risks of sudden cardiac death and cardiac dysfunction. The recently proposed “epilepsy–heart syndrome” concept captures the complex bidirectional relationship between epilepsy and cardiac disease. This concept includes both cardiac damage from chronic epilepsy and new‐onset epilepsy in patients with pre‐existing heart disease, providing a more comprehensive representation of the comorbidity burden [125]. It emphasizes the need for coordinated care through integrated heart–brain management.

Schachter et al. established multiscale diagnostic criteria for the epileptic heart, encompassing chronic epilepsy, electrocardiographic signs of myocardial injury or arrhythmia risk, autonomic dysfunction, as assessed by HRV, echocardiographic diastolic dysfunction, hyperlipidemia, and accelerated atherosclerosis [126]. ECG has substantial clinical value in epilepsy management. It demonstrates characteristic alterations across different seizure phases, making it useful for seizure prediction and risk assessment [112]. Specifically, preictal HRV analysis and QRS changes provide early warning; ictal monitoring identifies life‐threatening arrhythmias, such as tachycardia and asystole; and postictal repolarization abnormalities, such as QT prolongation, indicate electrical instability [112]. A multicenter Phase III trial validated a wearable HRV‐based ECG device, which achieved 90.5% sensitivity for detecting autonomic‐related seizures with low false‐positive rates. When combined with behavioral tests, it can also assess impaired awareness, demonstrating promising clinical utility [127]. In summary, the core clinical value of ECG in epilepsy management includes: (1) distinguishing SUDEP from primary arrhythmic death; (2) providing baseline screening for patients with newly diagnosed epilepsy and long‐term monitoring for high‐risk groups; and (3) enabling long‐term risk stratification through artificial intelligence‐assisted analysis and implantable monitors [112].

Electroencephalography (EEG) serves as a cornerstone in epilepsy diagnosis and management. The development of simultaneous EEG–ECG monitoring extends this capability, enabling comprehensive assessment of arrhythmia and SUDEP risk in patients with epilepsy [120]. Simultaneous ECG–EEG recording enables synchronous monitoring of neural and cardiac activity, helping identify cardiac comorbidities, including arrhythmias and autonomic dysfunction [128]. Advancing this understanding, a stereo‐electroencephalography study of 54 patients with drug‐resistant epilepsy mapped the spatiotemporal dynamics of heartbeat‐evoked potentials throughout the brain. This study confirmed that heartbeat‐evoked potentials involve not only established regions, such as the insula and anterior cingulate cortex, but also higher‐order areas such as the dorsolateral prefrontal cortex, identifying two primary components, early and delayed, mediated mainly by 5‐HT2A receptors [129]. In summary, the multimodal EEG‐ECG strategy captures autonomic imbalance signals in real time, with the potential to improve seizure prediction accuracy and improve early warning of sudden cardiac death [112].

Cardiac safety should be considered during antiseizure medication therapy, as certain agents can induce ECG abnormalities and arrhythmias through effects on ion channels [120]. A retrospective cohort study using the TriNetX database found that, compared with lamotrigine, carbamazepine (HR = 1.390) and valproic acid (HR = 1.264) were associated with an increased risk of composite cardiovascular events [130]. Routine ECG monitoring is therefore recommended during antiseizure medication initiation and dose titration [120]. For high‐risk patients, vagus nerve stimulation (VNS) offers an alternative therapy that improves cardiac electrical stability through autonomic modulation. This approach uses HR patterns for seizure monitoring and intervention, providing integrated heart–brain management for drug‐resistant epilepsy [112].

**FIGURE 2:**
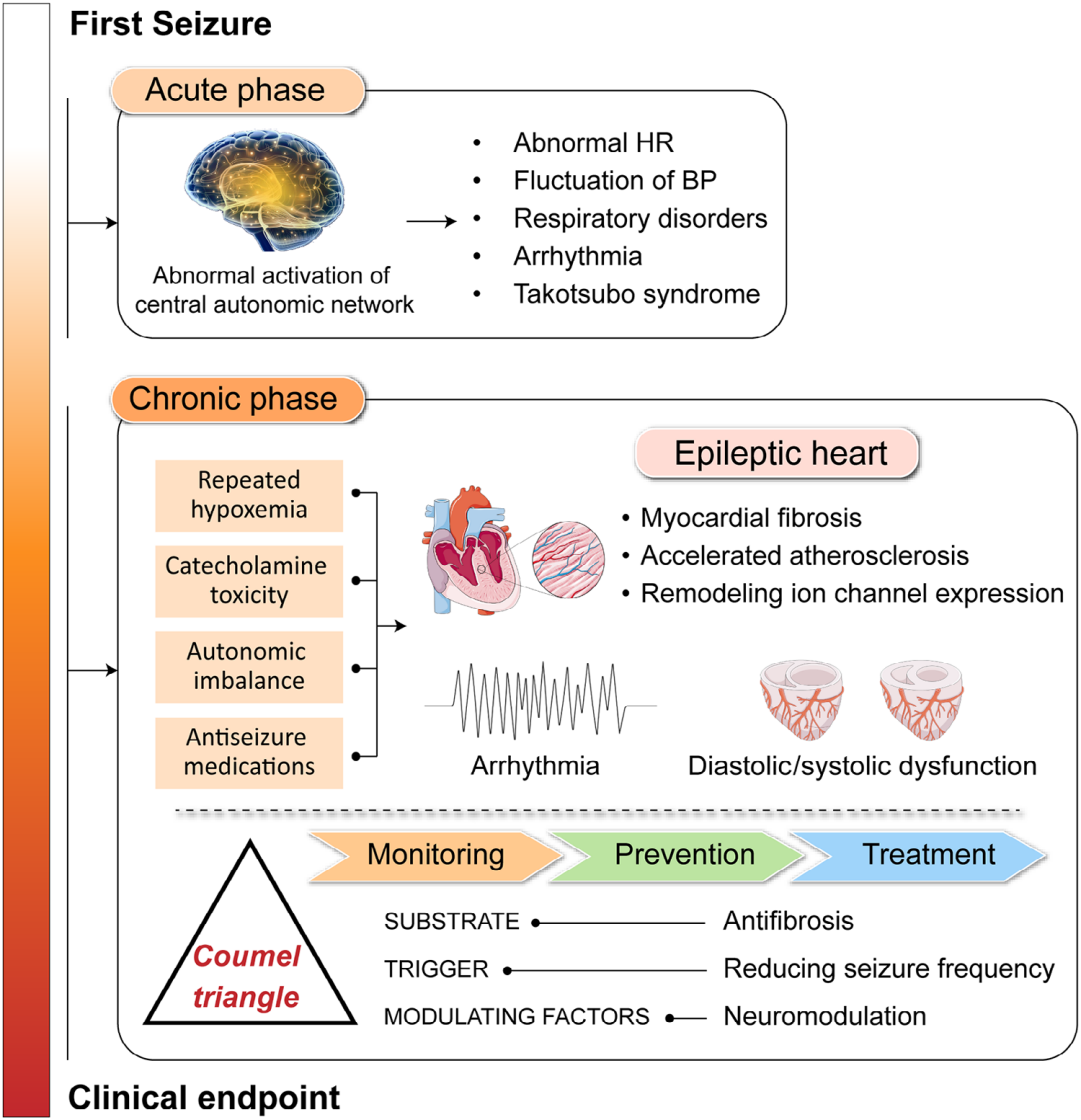
Heart–brain interactions in the epileptic heart. Acute seizures may activate the central autonomic network and induce transient cardiovascular disturbances, including abnormal heart rate, blood pressure fluctuations, respiratory dysfunction, arrhythmia, and Takotsubo syndrome. Recurrent seizures may contribute to chronic hypoxemia, catecholamine toxicity, autonomic imbalance, and antiseizure medication‐related cardiac effects, potentially contributing to myocardial fibrosis, accelerated atherosclerosis, ion channel remodeling, arrhythmias, and ventricular dysfunction. These changes are consistent with Coumel's arrhythmogenic triangle, supporting integrated strategies that combine cardiac monitoring, seizure prevention, antifibrotic therapy, and neuromodulation. BP, blood pressure; HR, heart rate.

Advancing genomic sequencing technologies now enable investigation of genetic associations between epilepsy and arrhythmias, offering opportunities to investigate the mechanisms underlying heart–brain channelopathy comorbidity [120]. SUDEP prevention requires a comprehensive approach. While current evidence does not support cardiac pacing alone for SUDEP prevention, treatment should prioritize optimization of antiseizure medications [120], future cardiac‐targeted strategies may have value. According to Coumel's triangle of arrhythmogenesis, epilepsy exhibits all three requisite elements: structural substrate, including myocardial fibrosis and remodeling; triggers, including ictal ischemia and catecholamine surge; and modulating factors, including autonomic imbalance [117]. This framework supports a three‐tiered intervention strategy: (1) improving cardiac structure via antifibrotic therapy; (2) optimizing antiseizure regimens to control seizure frequency; and (3) restoring autonomic balance through neuromodulation. Together, these strategies provide a translational framework linking pathogenesis to clinical intervention (Figure 2).

### 3.3 Stroke–Heart Syndrome

According to GBD 2021 data, there were 93.8 million prevalent cases of stroke and 11.9 million incident cases worldwide in 2021 [131]. Of these, ischemic stroke accounted for approximately 7.804 million new cases each year, representing about 65% of all incident strokes [132]. Against this substantial disease burden, stroke‐related complications, particularly cardiac complications, are major determinants of short‐term mortality and long‐term prognosis. In the first few days after acute ischemic stroke (AIS), patients may develop a range of cardiac manifestations, including myocardial injury, cardiac dysfunction, and arrhythmias [133]. These stroke‐related cardiac abnormalities share common pathophysiological features. They can therefore be collectively grouped under a distinct clinical entity, termed SHS [133]. These stroke‐related cardiac abnormalities were first described in the 1950s and 1960s, whereas the concept of “stroke–heart syndrome” was formally proposed by Scheitz et al. in 2018 [133]. SHS has a complex clinical spectrum. Its main manifestations include acute myocardial injury, ACS, left ventricular systolic or diastolic dysfunction, TTS, electrocardiographic abnormalities, arrhythmias, and sudden cardiac death. These phenotypes often overlap substantially [134]. These cardiac changes usually peak within the first 3 days after AIS [134, 135]. Cardiac abnormalities occurring within 30 days after stroke are generally considered part of SHS [134, 135].

#### 3.3.1 Epidemiology of SHS

A retrospective analysis of the VISTA database included 15,054 patients with AIS. The analysis showed that 11.8% of patients developed SHS, with a median time to onset of 2 days [135]. Among the specific phenotypes, arrhythmias and electrocardiographic abnormalities were the most common, with an incidence of 6.5% [135]. Another retrospective cohort study enrolled 365,383 patients with first‐ever ischemic stroke with a follow‐up period of 5 years. The incidences of specific poststroke cardiac complications varied markedly. ACS occurred in 11.1% of patients, AF or flutter in 8.8%, HF in 6.4%, severe ventricular arrhythmias in 1.2%, and TTS in 0.1% [136]. Overall, more than 27% of patients developed cardiac complications within 4 weeks after stroke [136].

SHS is closely associated with poor functional outcomes. SHS‐related cardiac complications are also the second leading cause of death in the first few weeks after stroke [133]. Studies have shown that, among patients with newly diagnosed poststroke cardiovascular complications, the 5‐year risk of recurrent stroke exceeds 50% [136]. In addition, SHS is associated with a higher risk of death, recurrent stroke, and acute MI within 5 years [137]. Notably, the timing of SHS onset may also be associated with prognosis. Patients with delayed‐onset SHS, defined as SHS occurring 10–30 days after stroke, are associated with a higher risk of death [135]. A retrospective cohort study of 21,931 patients with first‐ever AIS used propensity score matching to compare these patients with individuals without stroke. The study found that the risk of major adverse cardiovascular events was highest during the first 30 days after stroke, with HR = 25.0. Although the risk declined over time, it remained elevated from Days 31 to 90, with HR = 4.8, and from Days 91 to 365, with HR = 2.2 [138]. However, it remains unclear whether this long‐term increase in risk is mainly driven by stroke‐induced cardiac injury, pre‐existing subclinical CVD, or both. Further investigation is needed.

The risk of SHS may differ by sex. A retrospective study of 300 patients found that, among patients with AIS and elevated baseline cardiac troponin levels, women had approximately twice the risk of poststroke acute myocardial injury compared with men [139]. However, another retrospective study including 682,203 patients showed no significant difference in the incidence of SHS between men and women [137]. These findings suggest that current evidence on sex‐related differences remains inconsistent. Further studies are needed to clarify the association between sex and the risk of SHS.

#### 3.3.2 Pathophysiological Mechanisms of SHS

**FIGURE 3:**
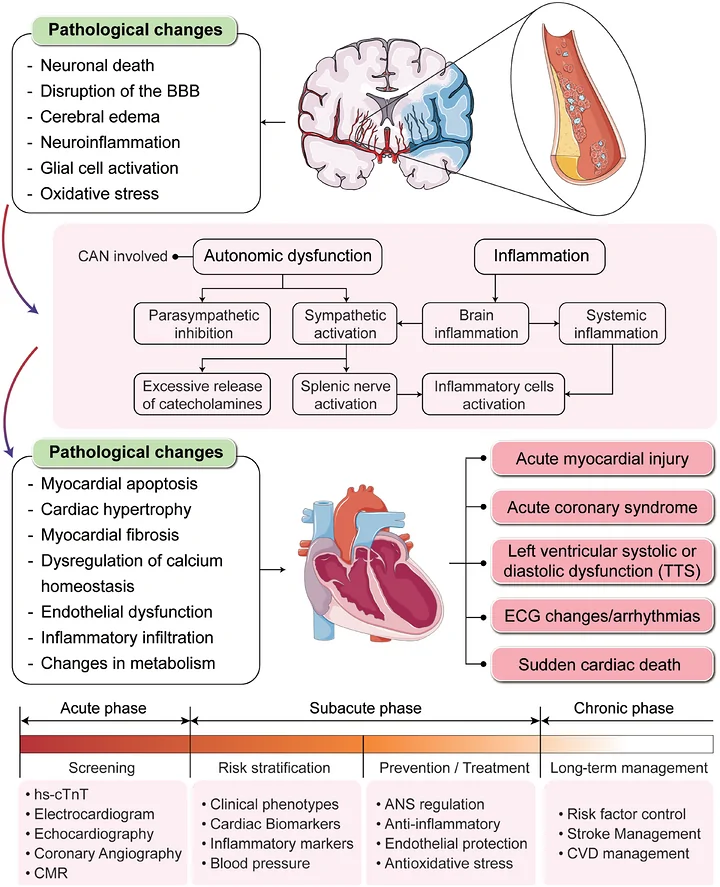
Heart–brain interactions in stroke–heart syndrome. Acute stroke can cause brain injury, including blood–brain barrier disruption, neuroinflammation, glial activation, oxidative stress, and neuronal death. When the central autonomic network is involved, stroke may induce autonomic dysfunction with sympathetic activation and parasympathetic suppression. Poststroke neuroinflammation may further enhance sympathetic outflow, promoting catecholamine release and splenic nerve activation. In parallel, neuroinflammation may develop into systemic inflammatory response. Together, these neuroimmune processes promote peripheral immune cell activation and contribute to cardiac injury, including myocardial apoptosis, hypertrophy, fibrosis, calcium dysregulation, endothelial dysfunction, inflammatory cell infiltration, and metabolic alterations. These pathological changes contribute to acute myocardial injury, acute coronary syndromes, ventricular dysfunction, ECG abnormalities, arrhythmias, and sudden cardiac death. Integrated management may include early cardiac screening, risk stratification, targeted prevention and treatment, and long‐term control of stroke and cardiovascular risk factors. ANS, autonomic nervous system; BBB, blood–brain barrier; CAN, central autonomic network; CMR, cardiac magnetic resonance; CVD, cardiovascular disease; ECG, electrocardiography; hs‐cTnT, high‐sensitivity cardiac troponin T; TTS, Takotsubo syndrome.

Overall, most stroke‐related cardiac abnormalities are potentially reversible. However, some patients still experience poor short‐term or even long‐term outcomes. Stroke‐induced functional and structural alterations in the CAN are currently considered a key pathophysiological basis of SHS [133]. Accordingly, SHS may serve as an important clinical model for understanding how brain injury affects cardiac function through neural–humoral–immune networks (Figure 3).

##### 3.3.2.1 Autonomic Dysfunction

The CAN provides an important anatomical basis linking brain injury to cardiac dysfunction. When stroke lesions involve brain regions within the CAN, they may disrupt the balance between sympathetic and parasympathetic tone. During the acute phase of AIS, autonomic balance often shifts toward sympathetic predominance. This shift is typically reflected in reduced HRV and baroreflex sensitivity [140]. Excessive activation of the sympathoadrenal axis may lead to excessive catecholamine release and is considered a key mechanisms underlying SHS. At the same time, reduced parasympathetic activity is also a major feature of autonomic imbalance in both the acute and chronic phases of cerebral ischemia [141]. Mouse models have shown that transcutaneous auricular VNS can enhance parasympathetic activity and attenuate sympathetic predominance [141]. At the phenotypic level, this intervention improves left ventricular ejection fraction (LVEF), reduces cardiomyocyte apoptosis, and alleviates myocardial hypertrophy and fibrosis [141]. Further mechanistic studies suggest that its cardioprotective effects may involve activation of muscarinic acetylcholine receptors, modulation of the PI3K–Akt pathway, and regulation of endothelial nitric oxide synthase [141].

As discussed above, autonomic regulation shows hemispheric lateralization. The right hemisphere predominantly regulates sympathetic regulation, whereas the left hemisphere is mainly involved in parasympathetic regulation [18]. The insular cortex is an important component of the CAN and plays an important role in cardiovascular autonomic control. Stroke location, especially involvement of the right insula cortex, is associated with the subsequent extent of cardiac injury and dysfunction [133]. A retrospective imaging cohort study of 231 patients with AIS showed that reduced LVEF was associated with acute lesions in the right insula, amygdala, hippocampus, and opercular region [142]. These findings suggest that damage to specific autonomic regulatory brain regions may constitute a neuroanatomical basis for SHS. Stroke severity is also associated with the development of SHS. A prospective study showed that stroke severity was an independent predictor of poststroke arrhythmias [143]. Mechanistic studies further suggest that poststroke autonomic imbalance may contribute to arrhythmogenesis through abnormal ion channel activity and cholinergic signaling [144]. In a rat model of stroke, abnormal activation of M_3_ receptor‐mediated cholinergic synaptic signaling and L‐type calcium channel‐mediated Ca^2^^+^ signaling was identified as a key mechanism underlying AF after ischemic stroke. Inhibition of this pathway partially reversed stroke‐induced AF [144].

A previous review summarized the local mechanisms through which stroke‐induced autonomic dysfunction contributes to myocardial injury [5]. After disruption of autonomic pathways, excessive sympathetic activation can lead to substantial accumulation of catecholamines in the myocardial interstitial space, whereas circulating catecholamine levels may increase only mildly [5]. This phenomenon may reflect the close proximity between sympathetic nerve terminals and cardiomyocytes [5]. Sympathetic nerve fibers are also heterogeneously distributed within the heart [5]. Among cardiac chambers, the left atrium has the densest sympathetic innervation. After stroke, the left atrium may therefore show more pronounced endothelial dysfunction, inflammation, and fibrosis [5]. At the cellular level, NE can exert direct toxic effects on cardiomyocytes by altering β‐adrenergic signaling. This may lead to contraction band necrosis, apoptosis, and uncoupling of myocardial β‐adrenergic receptors from downstream mechanical responses, thereby impairing contractile function [5]. NE can also increase myocardial oxygen demand, promote coronary atherogenesis, induce endothelial dysfunction, and increase plaque vulnerability. These effects may increase the risk of ACS in both the short and long term [5]. In addition, autonomic imbalance interacts with inflammation. Excessive sympathetic activation can promote the production of inflammatory mediators, thereby further contributing to pathological myocardial remodeling and arrhythmogenesis [5].

##### 3.3.2.2 Inflammation

Local brain injury after stroke can rapidly induce neuroinflammation, which may further trigger a systemic inflammatory response. One study using a brain‐targeted drug delivery system in a mouse model of stroke found that cerebral inflammation was markedly enhanced after stroke, followed by increased circulating levels of IL‐1β and ATP [63]. Neuroinflammation may also induce excessive sympathetic activation. Together, elevated inflammatory mediator levels and sympathetic activation may contribute to cardiac injury [63]. Studies in rat models of stroke have also shown that ischemic stroke is associated with increased circulating NE and epinephrine levels, accompanied by inflammatory cell infiltration and mitochondrial injury in the myocardium [145]. Further analysis showed increased macrophage recruitment in the heart after stroke, upregulation of ZBP1 and NLRP3, and enhanced production of inflammatory cytokines and activation of inflammasomes [145]. These findings suggest that poststroke cardiac injury may involve local inflammasome activation and immune cell infiltration in the myocardium. Transcriptomic analyses further support the activation of cardiac immune and inflammatory pathways after stroke. In mouse hearts after ischemic stroke, immune and inflammatory pathways were significantly enriched. Five differentially expressed genes, *Aplnr*, *Ccrl2*, *Cdkn1a*, *Irak2*, and *Serpine1*, were further identified as being associated with immune cell infiltration [146]. Clinical studies also suggest that inflammatory markers may have predictive value for SHS. A retrospective analysis based on the TriNetX database included 104,741 patients with AIS. It showed that C‐reactive protein levels measured within 24 h after stroke were associated with the risk of cardiac complications or death within 30 days [147].

Inflammatory responses and autonomic dysfunction are closely linked [5, 63]. Previous studies have proposed that spleen may serve as a key immune organ linking sympathetic activation, systemic inflammation, and cardiac injury [5]. Nearly 98% of splenic nerve fibers are noradrenergic sympathetic fibers. Inflammatory cells, including neutrophils, lymphocytes, monocytes, and macrophages, express β‐adrenergic receptors [5]. Therefore, increased sympathetic tone after stroke may exacerbate systemic and splenic inflammation. This may promote the release of splenic monocytes, which subsequently migrate to the heart and contribute to cardiac inflammatory cell infiltration and myocardial injury [5]. This “brain–spleen–heart” inflammatory axis provides an important framework for understanding how poststroke neuroinflammation may contribute to cardiac injury.

Furthermore, metabolic abnormalities may also contribute to the development of SHS. A Mendelian randomization study showed that Type 2 diabetes and lipid metabolic disorders may mediate the causal relationship between ischemic stroke and arrhythmias [148]. The same study also suggested that the cAMP signaling pathway and genetic variation in *PDE3A* may contribute to the development of arrhythmias in patients with ischemic stroke [148]. Consistent with these findings, a metabolomic study in rat models showed remodeling of plasma lipid metabolism in the plasma after stroke [149]. In cardiac tissue, the metabolic profile was characterized by increased collagen turnover, increased arginase activity and reduced nitric oxide synthase activity, and altered amino acid metabolism, including changes in pathways involving serine, asparagine, lysine, and glycine [149].

#### 3.3.3 Integrated Heart–Brain Management of SHS

Given that approximately one‐fifth of patients with AIS develop SHS, prevention, early recognition, and dynamic monitoring are essential. In patients with stroke, assessment of high‐sensitivity cardiac troponin levels, ECG, and echocardiography can help identify early cardiac involvement after ischemic stroke [134]. When necessary, coronary angiography and cardiac MRI may be performed to identify underlying coronary artery disease and characterize myocardial injury [134]. Biomarkers of cardiac stress, such as natriuretic peptides, may also indicate increased cardiac workload. Corrected QT (QTc) interval prolongation, arrhythmia, autonomic dysfunction, and elevated inflammatory markers should also be incorporated into poststroke cardiac risk assessment. A retrospective cohort study of 448 patients showed an interaction between QTc prolongation and elevated BNP levels in relation to short‐term all‐cause mortality in patients with AIS and SHS [150]. QTc prolongation was associated with an increased risk of mortality, especially in patients with elevated BNP levels [150]. Peripheral inflammatory markers, such as C‐reactive protein, may also have predictive value for the risk of SHS and subsequent prognosis [147]. Poststroke arrhythmia monitoring is also important. A single‐center prospective study of 335 patients with AIS found that 14% developed arrhythmias and 8% developed severe heart rhythm disturbances [151]. Severe heart rhythm disturbances were associated with increased disability at 3 months, new‐onset AF, and myocardial fibrosis, with ORs of 2.9, 3.5, and 5.8, respectively [151]. These findings suggest that poststroke arrhythmias may be related to subsequent functional recovery and structural myocardial changes.

The clinical manifestations of SHS are complex and highly heterogeneous. Risk prediction models that integrate clinical features, laboratory markers, and imaging information may facilitate the identification of high‐risk patients. A retrospective study of 218 patients with AIS who underwent endovascular treatment developed a prediction model for SHS using age, hyperlipidemia, creatinine levels, and total anterior circulation infarction [152]. The model showed good discriminative performance, with an AUC of 0.812 [152]. Clinical phenotype‐based stratification may also support individualized management. One study performed hierarchical cluster analysis in 12,482 patients with AIS from the VISTA database. Five clinical profiles were identified: Profile 1, “older patients with AF”; Profile 2, “younger smokers”; Profile 3, “younger patients”; Profile 4, “cardiac comorbidity”; and Profile 5, “hypertension with atherosclerosis.” [153] Among these profiles, Profile 4 and Profile 1 had the highest risk of SHS, with HRs of 2.01 and 1.26, respectively [153]. Profile 5 had the highest risk of cardiac arrest, with an HR of 2.99 [153]. Profiles 5 and 4 also had the highest 90‐day mortality [153]. Therefore, recognition of these clinical profiles may support SHS risk management. BP is another important factor in SHS risk stratification. A retrospective analysis of 14,965 patients from the VISTA database showed a U‐shaped relationship between initial BP levels and the risk of SHS [154]. At higher BP levels, the risk of cardiopulmonary arrest was greatest. At lower BP levels, the risk of other SHS manifestations was higher [154]. These findings suggest that BP management in the acute phase of stroke may need to consider not only cerebral perfusion and neurological recovery but also the risk of cardiac complications.

Reperfusion therapy for AIS may be associated with the risk of SHS, but current findings are not fully consistent. A study of 120 patients with AIS due to anterior circulation occlusion found that patients who received intravenous thrombolysis before thrombectomy had lower peak cardiac troponin and BNP levels than those who underwent direct thrombectomy. They also had lower modified Rankin Scale scores at discharge [155]. Similarly, another retrospective study of 411 patients suggested that reperfusion therapy may be associated with a lower risk of SHS in patients with AIS due to total anterior circulation occlusion [156]. However, a retrospective cohort study of 76,092 patients found that patients with AIS who underwent mechanical thrombectomy had a higher risk of cardiac events than those treated with intravenous thrombolysis or those receiving no reperfusion therapy, with cause‐specific HRs of 1.48 and 1.39, respectively [157]. This discrepancy may be related to differences in baseline stroke severity, the extent of vascular occlusion, treatment indications, and periprocedural stress responses. Therefore, the relationship between reperfusion therapy and SHS requires further clarification in prospective studies with more rigorous control of confounding factors.

In addition, the development and prognosis of SHS depend not only on stroke itself but also on pre‐existing cardiovascular risk factors. Among patients with SHS, those with additional cardiovascular risk factors have the highest risk of adverse outcomes. These factors include hypertension, Type 2 diabetes, elevated low‐density lipoprotein cholesterol, and a history of smoking [137]. Therefore, active management of these risk factors may improve long‐term prognosis. For patients with stroke, integrated heart–brain management should not be limited to the identification of cardiac complications in the acute phase. It should also include long‐term control of BP, blood glucose, and blood lipids, smoking cessation, and appropriate use of antiplatelet, anticoagulant, or lipid‐lowering therapies. These strategies may reduce the risk of recurrent stroke, MI, and death.

Based on the underlying pathophysiology, potential future therapeutic strategies may include targeting the ANS through β‐blockers or VNS; optimizing HR control and reducing BP variability; using anti‐inflammatory therapies; improving vascular endothelial function; reducing oxidative stress; optimizing the combined use of antithrombotic agents; and avoiding proarrhythmic drugs [134]. VNS has been proposed as a potential preventive and therapeutic strategy. However, a single‐center retrospective cohort study of 490 patients found that chronic prestroke treatment with cardioselective β_1_‐blockers was not associated with a reduced risk of SHS [158]. This finding suggests that β_1_‐adrenergic blockade alone may be insufficient to address the complex pathophysiology of SHS. Future therapies may need to target autonomic–inflammatory interactions more precisely.

At present, most studies of SHS are retrospective. Large‐scale multicenter prospective studies are needed to further clarify the relationships among stroke location, stroke severity, autonomic function, inflammatory responses, and specific cardiac phenotypes. Such studies are also needed to evaluate and validate preventive and therapeutic strategies for SHS. A major future challenge is to develop SHS‐specific treatments that improve long‐term outcomes.

### 3.4 Heart Failure

**FIGURE 4:**
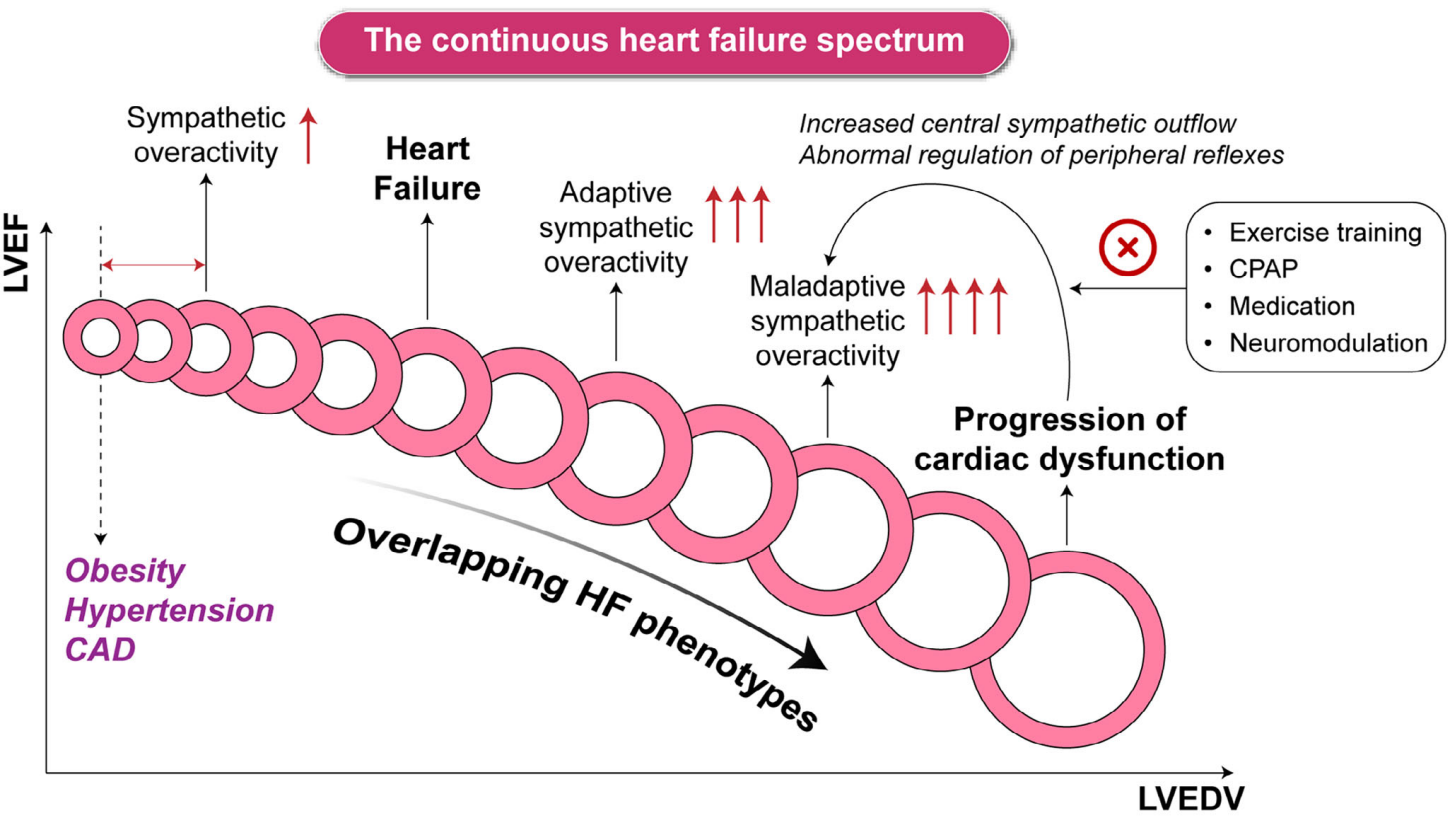
Heart–brain interactions in heart failure. Sympathetic activation evolves across the heart failure continuum. In the early stage, comorbidities such as obesity, hypertension, and coronary artery disease may trigger adaptive sympathetic overactivity as left ventricular function declines. With disease progression, sustained central sympathetic outflow and abnormal peripheral reflex regulation transform this compensatory response into maladaptive sympathetic overactivity, thereby further worsening cardiac dysfunction and creating a self‐perpetuating heart–brain cycle. Interventions such as exercise training, CPAP, pharmacological therapy, and neuromodulation may help interrupt this cycle. CAD, coronary artery disease; CPAP, continuous positive airway pressure; HF, heart failure; LVEDV, left ventricular end‐diastolic volume; LVEF, left ventricular ejection fraction.

Autonomic imbalance, characterized by sympathetic overactivation and parasympathetic suppression, constitutes a core pathophysiological component of HF (Figure 4). HF has even been proposed to exhibit features of a “brain‐originated disorder” [159]. Elevated plasma NE in patients with HF has been identified as an independent prognostic indicator, with levels correlating positively with overall sympathetic activity [160]. A study employing isotope dilution in 60 patients with moderate‐to‐severe HF demonstrated that the cardiac NE spillover rate, which reflects local cardiac sympathetic activity, was associated with poor prognosis [161]. A subsequent large‐sample study found that vagal reflex sensitivity decreased with worsening cardiac function and was associated with poorer survival (HR = 2.7) [162]. Collectively, these findings underscore the central role of autonomic imbalance in CHF progression.

#### 3.4.1 Autonomic Dysfunction as an Initiating Factor in HF

The HF continuum theory posits that disease progression involves the dynamic evolution of cardiac function and structure, thereby forming a disease spectrum with distinct trajectories and overlapping phenotypes. Throughout this continuum, maladaptive neurohormonal activation is a common feature and becomes progressively more pronounced as LVEF declines [163, 164]. Sympathetic activation persists throughout the disease course: initially serving as a compensatory response to cardiac injury, it progressively becomes maladaptive that ultimately contributes to cardiac decompensation [165]. Notably, this early sympathetic activation may be related to the sympathetic overactivity associated with risk factors such as obesity and hypertension [165].

Mechanistically, the Angiotensin II (Ang II) signaling pathway serves as a central link between autonomic imbalance and HF progression. Centrally, Ang II stimulates NE release and induces oxidative stress and inflammation through its actions in the PVN, RVLM, and area postrema. Peripherally, Ang II enhances neurotransmitter release from sympathetic nerve terminals while inhibiting neurotransmitter reuptake. By potentiating excitatory chemoreflexes and suppressing inhibitory baroreflexes, Ang II establishes a positive feedback loop that sustains autonomic overactivation [159]. This combined central and peripheral dysregulation may contribute to the pathogenesis and progression of HF.

MI is an important cause of HF. In randomized clinical trials of HF with reduced ejection fraction (HFrEF), 57.8% of patients were classified as having an ischemic etiology, and 46.7% had a history of MI [166]. HF is also a common complication after MI, occurring in approximately 13% of patients within 30 days and in 20–30% within 1 year [167]. A recent study identified a three‐node heart–brain neuroimmune circuit involving sensory afferent signaling, central integration, and sympathetic output [168]. The study showed that MI activates TRP vanilloid 1 (TRPV1)‐positive vagal sensory neurons, which transmitted cardiac injury signals to the brain and activate Ang II Type 1a receptor (AT_1_aR)‐positive neurons in the PVN. This response then enhances sympathetic output through the superior cervical ganglion and promoted IL‐1β‐mediated inflammation, thereby worsening postinfarction cardiac injury. Ablation of TRPV1‐positive vagal sensory neurons, inhibition of PVN AT1aR neurons, or blockade of IL‐1β signaling in the superior cervical ganglion all improved cardiac outcomes after MI [168]. These findings provide new evidence for understanding early sympathetic activation and its neuroimmune basis in post‐MI HF.

#### 3.4.2 HF Exacerbates Autonomic Imbalance: Forming a Vicious Cycle

##### 3.4.2.1 Increased Central Sympathetic Outflow

In HF, neuronal reuptake of NE is markedly impaired. Studies have demonstrated a 47% reduction in myocardial NE storage capacity and a 42% decrease in vesicle leakage rate in patients with HF compared with individuals without HF, indicating severely compromised NE reuptake and storage efficiency [169]. Radiotracer studies have further revealed a 277% increase in cardiac NE spillover rate in patients with HF accompanied by activation of central noradrenergic neurons [170]. Notably, increased noradrenergic activity in subcortical regions projecting to the brainstem positively correlates with systemic NE spillover, indicating that activation of rostrally projecting noradrenergic neurons may represent a key central mechanism contributing to sympathetic activation in HF [171].

Sympathetic activation in HF shows marked regional specificity. In mild‐to‐moderate HF, cardiac NE spillover is threefold higher than in healthy individuals, whereas total‐body and renal NE spillover and skeletal muscle sympathetic activity remain unchanged [172]. In severe HF, cardiac NE spillover increases fourfold, accompanied by elevated total‐body and renal NE spillover and increased skeletal muscle sympathetic activity [172]. This progressive escalation suggests preferential cardiac sympathetic activation associated with disease severity. Excessive cardiac sympathetic activation, impaired neuronal NE reuptake, and multifiber systolic sympathetic discharge constitute the “sympathetic toxicity triad,” a hallmark of “adrenergic toxicity” in HF [173].

HF with preserved ejection fraction (HFpEF) and HFrEF differ distinctly in ANS regulation. Patients with HFpEF show no clear evidence of cardiac sympathetic overactivity, and β‐blockers lack established prognostic benefit in this population [174]. Studies of HR regulation during exercise in HFpEF indicate preserved afferent metaboreflex pathways (sympathetically mediated) and central command feedforward pathways (vagally mediated) [175]. In contrast, patients with HFrEF exhibit pronounced sympathetic activation, with markedly increased sympathetic outflow to the heart, kidneys, and skeletal muscle vasculature [176]. Cardiac NE spillover rates are four‐ to sixfold higher in patients with HFrEF than in healthy individuals [161, 177], and this sympathetic overactivation is strongly associated with adverse outcomes [161].

HF is frequently complicated by respiratory and renal dysfunction, which may contribute to sodium and water retention and volume overload. In a salt‐loaded HFpEF rat model, activation of RVLM C1 neurons markedly enhanced cardiac sympathetic drive and exacerbated respiratory dysfunction. Conversely, selective ablation of these neurons attenuated sympathetic overexcitation, improved respiratory function, and reduced decompensation‐related mortality [178]. Thus, RVLM C1 neurons may contribute to acute HF decompensation during salt loading through enhanced sympathetic outflow and respiratory impairment. This process may ultimately disrupt cardiomyocyte calcium handling and impair cardiac contractility [178]. At the molecular level, mouse models of HF exhibit impaired mitochondrial function and elevated circulating DHODH levels. This alteration may activate cGAS signaling in the CAN, thereby exacerbating vascular inflammation, promoting glial activation and neuronal sensitization, and potentially worsening sympathetic overactivation and myocardial injury [179].

Collectively, these findings demonstrate that HF activates specific central neural pathways, driving sustained increases in sympathetic outflow and establishing a self‐perpetuating vicious cycle of heart–brain interactions.

##### 3.4.2.2 Altered Peripheral Sympathetic Inflow (Reflex Mechanisms)

Dysfunctional peripheral autonomic reflexes are closely linked to sympathetic activation in CHF [173]. Baroreflex dysfunction emerges early in HF, with impairment evident even in mild cases and progressive deterioration as the disease advances [180]. This early baroreflex impairment may contribute to sympathetic activation in patients with HF, contributing to a vicious cycle of disease progression. Mechanistically, the aortic baroreceptor discharge response is blunted in HF models, resulting in an elevated activation threshold [181]. Regardless of the specific mechanism, the net effect is attenuated baroreflex inhibition of central sympathetic output. Concurrently, cardiopulmonary baroreceptor desensitization further exacerbates sympathetic overactivation. In congestive HF, increased cardiac sympathetic activity correlates positively with elevated pulmonary artery pressure and pulmonary capillary wedge pressure [170]. Nitroprusside administration in patients with HF resulted in a concurrent increases in whole‐body NE spillover and decreases in cardiac NE spillover [182]. This inverse systemic–cardiac sympathetic relationship suggests combined impairment of arterial and cardiopulmonary baroreflexes [182]. This reflex imbalance may be closely related to chronic volume overload, with mechanisms analogous to microgravity‐induced sympathetic activation in astronauts [183].

Patients with HF frequently exhibit respiratory instability, including hypoventilation and apnea. Reduced arterial oxygen levels and elevated carbon dioxide levels stimulate carotid body chemoreceptors, potentiating sympathetic responses [184]. Consequently, chemoreflex sensitivity is heightened in patients with HF and is independently associated with mortality risk (HR = 2.8) [184]. In rat models of HF, heightened chemoreflex sensitivity is associated with abnormal episodic discharge from the carotid body. Sympathetic activation triggers intermittent ATP release from glomus cells, generating aberrant electrical signals [185]. Notably, purinergic P2×3 receptor expression in peripheral chemoreceptor afferents is approximately doubled. P2×3 receptor antagonism attenuates abnormal discharge, normalizes chemoreceptor sensitivity and breathing patterns, restores autonomic balance, and reduces inflammatory and HF‐related biomarkers [185].

Beyond cardiovascular reflexes, additional peripheral mechanisms, including thermoregulatory reflexes and skeletal muscle metaboreflexes, contribute to sympathetic overactivation in HF [173]. Concurrent baroreflex and chemoreflex abnormalities may collectively contribute to autonomic imbalance, reduced exercise tolerance, ventilatory instability, and HF progression. A study systematically evaluating baroreflex and chemoreflex sensitivity in 425 patients with CHF assessed their prognostic value [186]. Reduced baroreflex sensitivity was associated with decreased exercise capacity and HRV, whereas enhanced chemoreflex sensitivity was associated with elevated plasma NE levels and a higher frequency of central sleep apnea [186]. During a median 50‐month follow‐up, reduced baroreflex sensitivity was primarily associated with the risk of cardiac death or an implantable cardioverter‐defibrillator shock, whereas enhanced chemoreflex sensitivity was associated with the risk of HF hospitalization [186]. Patients with combined abnormalities had the poorest outcomes. After adjusting for standard prognostic factors, both reduced baroreflex sensitivity (HR = 2.76) and enhanced chemoreflex sensitivity (HR = 2.91) remained independently associated with adverse outcomes [186].

#### 3.4.3 Integrated Heart–Brain Management of HF

Exercise training and continuous positive airway pressure (CPAP) are effective strategies for improving autonomic function in HF. A meta‐analysis showed that exercise training improves HRV and reduces muscle sympathetic nerve activity in patients with HF [187]. This benefit may be mediated through by activation of preganglionic neurons in the nucleus ambiguus and dorsal motor nucleus of the vagus following long‐term exercise training [36]. Short‐term CPAP therapy reduced cardiac NE spillover, suggesting an immediate reduction in sympathetic activity [188]. In patients with HF and comorbid obstructive sleep apnea, nocturnal CPAP therapy reduces daytime sympathetic activity, systolic BP, and HR [189].

Pharmacological therapy represents an important approach to modulating autonomic balance in HF. β‐Blockers without intrinsic sympathomimetic activity, such as carvedilol and metoprolol, effectively reduce excessive cardiac sympathetic activation. ACE inhibitors and ARBs may reduce sympathetic activity through both central and peripheral mechanisms: centrally, by reducing sympathetic nerve firing; and peripherally, by decreasing NE spillover and enhancing tissue clearance. These agents may also improve baroreflex function [173]. Scintigraphic imaging studies suggest that spironolactone may exert beneficial effects by reducing myocardial sympathetic drive [190]. Centrally acting sympatholytic agents, such as clonidine, reduce circulating and cardiac NE levels [191]. However, a multicenter randomized trial yielded paradoxical findings for moxonidine: despite an 18.8% reduction in plasma NE levels, moxonidine was associated with poorer outcomes and more hospitalizations for adverse events, potentially because of off‐target effects resulting from its limited selectivity [192].

VNS has shown therapeutic potential in HF. A 2011 multicenter study showed that implantable cervical VNS improved quality of life, exercise tolerance, and cardiac function in patients with HF [193]. Subsequently, a randomized trial found that transcutaneous VNS enhanced both global longitudinal strain and quality of life in patients with HFpEF and reduced TNF‐α levels, suggesting that modulation of the neuro–immune–inflammatory axis may underlie these effects [194]. However, the INOVATE‐HF trial showed that although VNS improved symptoms and NYHA functional class, VNS did not improve left ventricular remodeling or mortality or morbidity endpoints [195]. Consequently, the long‐term efficacy of VNS requires further evaluation.

Baroreflex activation therapy uses electrical stimulation of carotid sinus baroreceptors to modulate autonomic function. Although a prospective study showed that baroreflex activation therapy did not reduce the occurrence of the primary endpoint in patients with HFrEF, it provided sustained improvements in symptoms and quality of life, with an acceptable safety profile [196]. A meta‐analysis showed that Barostim, the first Food and Drug Administration‐approved neuromodulation device for HFrEF, was associated with improvements in quality of life and cardiac function [197]; nevertheless, current evidence remains insufficient to support its inclusion in guideline recommendations [4].

Other neuromodulation approaches are also under investigation. Renal denervation, by reducing plasma renin activity, aldosterone levels, and central sympathetic drive, may represent a promising approach, particularly for patients with HF and hypertension [198]. Preliminary studies of right‐sided greater splanchnic nerve ablation in patients with HFpEF have reported improvements in quality of life, exercise tolerance, and NT‐proBNP levels, with a favorable safety profile [199]. In summary, these emerging neuromodulation technologies may represent promising approaches to HF treatment, although further evidence is needed to clarify their therapeutic roles.

The neuro–immune axis plays an important regulatory role in HF. At the peripheral level, sympathetic overactivation may promote myocardial fibrosis and oxidative stress through mechanisms including β_1_‐adrenergic receptor desensitization and GRK2 upregulation. In parallel, impaired parasympathetic function weakens the cholinergic anti‐inflammatory pathway, potentially exacerbating systemic inflammation. Centrally, microglial activation within the PVN and increased BBB permeability may establish a self‐perpetuating cycle of neuroinflammation, which may ultimately contribute to autonomic imbalance [200]. Consequently, therapeutic strategies targeting this axis, such as VNS and SGLT2 inhibitors, may have the potential to improve cardiac function [194, 200].

Despite the potential of neuromodulation to counteract autonomic imbalance in HF, its clinical translation faces three major challenges: insufficient large‐scale safety and efficacy data, poorly defined target populations, and a lack of personalized treatment protocols with optimized stimulation parameters.

### 3.5 Cardiac Dementia

Cardiac dementia refers to cognitive decline associated with cardiac conditions, including HF, MI, and AF [201]. This concept was first introduced in *The Lancet* in 1977 to describe CI associated with diverse cardiac conditions [201]. A 21‐year follow‐up study revealed that a greater burden of cardiovascular risk factors was independently associated with accelerated decline in global cognition, episodic memory, working memory, and processing speed. Notably, this cognitive decline was associated with structural brain alterations, such as reduced hippocampal volume, gray matter atrophy, and greater white matter hyperintensity volume [202]. Collectively, these findings support an association between cardiovascular risk factors and both neurodegenerative and vascular brain injury.

#### 3.5.1 Epidemiology of Cardiac Dementia

A well‐established epidemiological link exists between AF and CI. A large prospective cohort study reported an annual incidence of CI of 8.70 per 1000 person‐years among patients with AF, with Alzheimer's disease (69.0%), vascular dementia (21.8%), and other dementias (9.0%) constituting the main subtypes [203]. This association is further supported by a meta‐analysis, which showed that AF was associated with increased risk of CI (OR = 1.5), all‐cause dementia (OR = 1.6), vascular dementia (OR = 1.7), and Alzheimer's disease (HR = 1.4) [204]. Long‐term follow‐up data indicate that AF was associated with a nearly threefold higher risk of dementia over 12 years, an association that remained evident even after excluding individuals with stroke [205]. Notably, 24.6% of patients with AF had CI before any ischemic stroke or transient ischemic attack [206]. Furthermore, AF is associated with a 2.7‐fold higher risk of poststroke CI, with information processing speed being the most affected domain [207]. Even among older adults without AF, frequent premature atrial complexes are independently associated with declines in executive function (*β* = −0.30) and an increased dementia risk (OR = 1.74), suggesting that abnormal atrial electrical activity may represent a potential biomarker for CI [208].

Independent of AF, HF is strongly associated with CI. Although the reported prevalence of CI among patients with HF varies widely (3–80%), the risk of CI remains elevated compared with the general population (HR = 1.3–1.7) [209]. Meta‐analyses indicate a pooled prevalence of CI of approximately 41–43% among patients with HF, with the prevalence of dementia reaching 19.79% [210, 211]. A Danish nationwide cohort study showed that the risk of all‐cause dementia among patients with HF increases cumulatively over time, with the risk of vascular dementia being particularly elevated (HR = 1.49) [212]. Furthermore, HF severity is correlated with the degree of cognitive decline, as both NYHA functional Class II or higher and reduced LVEF are independently associated with CI [213, 214].

Beyond the conditions previously discussed, several other cardiac diseases are linked to an elevated risk of CI. A meta‐analysis involving over 3 million subjects showed that coronary artery disease was associated with a relative risk of dementia of 1.27 [215]. Notably, nearly half (48.0%) of older patients presenting with non–ST‐segment elevation ACS had previously undiagnosed cognitive dysfunction [216]. Furthermore, patients with dilated or hypertrophic cardiomyopathy may exhibit distinct patterns of cognitive deficits and an increased risk of dementia [217, 218].

#### 3.5.2 Pathophysiological Mechanisms of Cardiac Dementia

##### 3.5.2.1 Cerebral Hypoperfusion and Impaired Blood Flow Regulation

In HF, particularly HFrEF, chronic cerebral hypoperfusion is considered a key mechanism underlying CI [219]. This cascade begins with reduced cerebral blood flow, which may trigger oxidative stress and neuroinflammation, characterized by elevated levels of inflammatory mediators, including elevated TNF‐α and IL‐6 levels, microglial activation, and mitochondrial dysfunction. The ensuing persistent hypoperfusion may further induces synaptic dysfunction and neuronal apoptosis, with pronounced effects in hypoxia‐sensitive regions such as the hippocampus and cortical layers III and V [219]. Notably, chronic hypoperfusion may also contribute to Alzheimer's disease‐like pathology, characterized by increased amyloid‐β (Aβ) production and tau protein hyperphosphorylation [219].

Data from the AGES sub‐study demonstrated that each 1 L/min decrease in cardiac output was associated with a 3.9‐mL reduction in total brain parenchymal volume in patients with HF [220]. Furthermore, each 10‐mL decrease in left ventricular stroke volume and each 1.0 L/min decrease in cardiac output were associated with 24 and 40% higher risks of mild CI or dementia, respectively [220]. Imaging studies further showed that the degree of medial temporal lobe atrophy correlated with the severity of CI in patients with HF [221]. The MATTERS‐HF cohort study found that reduced baseline hippocampal volume was associated with impaired cognitive function even in patients with mild HF [222]. Notably, early intervention for low cardiac output may improve cognitive dysfunction in older patients with HF [223].

However, the relationship between cardiac function and cognitive decline is not straightforward. A prospective cohort study found no association between the rate of cognitive decline and LVEF in patients with HF [224]. The ARIC study similarly found no difference in the incidence of CI between patients with HFrEF and those with HFpEF [225]. This apparent discrepancy may be explained by cerebrovascular regulatory mechanisms. Cerebral autoregulation normally maintains relatively constant blood flow across a wide range of perfusion pressures, but this compensatory mechanism may itself be impaired in HF [226]. Consequently, cerebral hypoperfusion combined with dysregulated blood flow control may leads to insufficient cerebral oxygen and nutrient delivery and neuronal damage [226].

Impaired cerebral hemodynamics represents another key mechanism underlying CI in patients with AF. Near‐infrared spectroscopy has revealed reduced hemodynamic responses in the frontal and temporal lobes of patients with persistent AF. Catheter ablation may restore hemodynamic responses in these regions, with recovery in the temporal lobe strongly correlating with cognitive improvement (*r* = 0.72) [227]. Neurovascular coupling is also impaired in patients with AF, as evidenced by visual stimulation tests showing a delayed hemodynamic response and diminished autoregulatory capacity [228]. Accordingly, patients with AF exhibit a 16% reduction in global cerebral perfusion and a 31% reduction in cerebrovascular CO_2_ reactivity compared with healthy controls, indicating limited cerebrovascular reserve [229]. A proposed mechanism is that AF‐related chronic hypoperfusion may trigger persistent compensatory arteriolar dilation, which exhausts vasodilatory reserve and disrupts cerebral autoregulation. Consequently, these disturbances may compromise BBB integrity, increase the susceptibility of brain tissue to ischemic injury, and thereby increase the risk of dementia and worsen stroke outcomes [230].

##### 3.5.2.2 Embolic Cerebrovascular Events and Cerebral Microbleeds

Both AF and HF are associated with an increased risk of stroke, primarily through cardiogenic embolism. This thromboembolic process involves the components of Virchow's triad, encompassing hypercoagulability, endothelial dysfunction, and blood stasis [231]. In HF, coagulation abnormalities are evident, with elevated markers of platelet activation and coagulation activity, alongside impaired fibrinolysis [231, 232]. Endothelial dysfunction is also prominent, as indicated by elevated levels of markers of endothelial injury and activation [233]. Left ventricular dilation and systolic dysfunction contribute to intracardiac blood stasis, which may be exacerbated by concomitant AF or valvular disease. Furthermore, in patients with ischemic HF, systemic atherosclerosis is linked to an increased risk of large artery atherosclerotic stroke, while a low cardiac output state may predispose patients to hemodynamic stroke, particularly in the presence of carotid or cerebral artery stenosis [231].

AF and dementia share numerous risk factors, including hypertension, diabetes, HF, smoking, vascular disease, sleep‐disordered breathing, and older age. Collectively, these risk factors may contribute to neuropathological changes, including white matter hyperintensities, silent brain infarcts, hippocampal atrophy, reduced gray matter volume, and functional neural network abnormalities, which may ultimately contribute to cognitive decline [219]. These cerebral neuropathological changes may therefore contribute to CI in patients with AF.

AF is associated with an increased risk of stroke through mechanisms consistent with Virchow's triad: hemodynamic disturbances, a hypercoagulable state, and endothelial dysfunction [234, 235]. Patients with poststroke AF have a 2.70‐fold higher risk of CI [236]. Even without a clinical stroke history, patients with AF exhibit structural brain abnormalities. Specifically, CI in AF is independently associated with cerebral small vessel disease, manifested by lacunes and white matter hyperintensities, and neurodegenerative changes such as medial temporal lobe atrophy [206]. Neuroimaging studies further support these associations. A meta‐analysis showed associations between AF and cerebral small vessel disease, including white matter hyperintensities and cerebral microbleeds (OR = 1.38), as well as silent brain infarction (OR = 2.11) [236]. MRI studies showed a higher prevalence of lacunar infarction (OR = 2.7) and silent brain infarction (OR = 3.5) among patients with AF [237]. Furthermore, multimodal neuroimaging has revealed extensive abnormalities in patients with AF and CI, encompassing cortical thinning, reduced gray matter volume, increased extracellular free water, and impaired white matter integrity [238]. Collectively, these findings support a potential mediating role of structural brain changes in AF‐related cognitive dysfunction and highlight the potential diagnostic value of neuroimaging biomarkers.

##### 3.5.2.3 Neurodegeneration

HF and neurodegenerative processes are closely linked at the molecular level. Patients with cardiac dysfunction exhibit altered serum levels of neurodegeneration biomarkers, including phosphorylated tau, neurofilament light chain, and Aβ_40_ [219]. Neuroimaging studies have revealed disrupted functional connectivity in the precuneus of patients with HF, a brain region affected early in Alzheimer's disease [219]. Genetic evidence further indicates that variants in Alzheimer's disease‐related genes, including *PSEN1* and *PSEN2*, are associated with an increased risk of HF. Conversely, Aβ deposition accompanied by diastolic dysfunction has been observed in the myocardium of patients with Alzheimer's disease [219].

Recent experimental studies demonstrate that HF increases cerebral arterial pulsatility in mice, thereby increasing glymphatic influx and subsequently inducing glymphatic dysfunction [239]. Since the glymphatic system clears metabolic waste from the brain, dysfunction of this system may promote protein aggregation and thereby accelerating neurodegeneration.

In AF‐related CI, cerebral hypoperfusion has been proposed to contribute to neurodegeneration through two pathway: it may promotes Aβ production by enhancing β‐secretase activity, impairs Aβ clearance, and directly activate tau kinase pathways [240]. However, existing neuropathological studies have not yet established a direct causal link between classical Alzheimer's disease pathology and AF.

##### 3.5.2.4 Inflammation

Conditions such as HF, coronary artery disease, and AF may lead to chronic activation of the NLRP3 inflammasome, thereby promoting the release of proinflammatory cytokines. These circulating cytokines may, in turn, compromise the BBB integrity, activate microglia, and trigger neuroinflammation. Collectively, this systemic and neuroinflammatory cascade may not only exacerbate cerebrovascular injury but also contribute to key pathological hallmarks of Alzheimer's disease: Aβ deposition and tau hyperphosphorylation [241].

In AF, systemic inflammation contributes to cognitive decline through several distinct pathways. First, inflammatory mediators, including IL‐1β and TNF‐α, may induce endothelial dysfunction and activate coagulation, thereby contributing to cerebrovascular injury. Second, the inflammatory microenvironment may promote atrial electrical and structural remodeling, which may contribute to AF persistence. Concurrently, microglial activation in the brain and upregulated β‐secretase expression may increase Aβ production. These processes may establish a self‐perpetuating cycle linking systemic inflammation, AF persistence, and neurodegeneration [219].

Similarly, CI in HF involves inflammatory and oxidative stress mechanisms. Animal studies have shown that CHF may activate the TLR4–TNF‐α–IL‐6 signaling pathway in the brain, resulting in dysregulated Aβ metabolism [242]. Furthermore, HF may upregulate E‐selectin in cerebral endothelial cells, thereby compromising BBB integrity, promoting cortical oxidative stress and Aβ deposition, and potentially contributing to vascular CI [243]. In rat models, circulating Ang II may enter the CNS, bind to AT_1_a receptors on hippocampal microglia, and trigger a neuroinflammatory cascade that culminates in CI [244]. Additionally, extracellular vesicles released following cardiac injury transport molecules such as microRNA‐21 into the brain, potentially promoting microglial polarization toward a proinflammatory M1 phenotype and contributing to neuroinflammation and cognitive deficits [245]. Collectively, these findings support an important role for inflammatory mechanisms in HF‐related CI.

#### 3.5.3 Integrated Heart–Brain Management of Cardiac Dementia

Clinical screening in patients with cardiac disease should follow a bidirectional assessment framework. Prospective studies suggest that cognitive function may influence treatment outcomes in HF. Therefore, pretreatment cognitive screening may guide the development of personalized management plans tailored to patients’ cognitive status [246]. Accordingly, van Nieuwkerk et al. proposed a stepped‐care strategy for CI screening in this population [247]. To facilitate this approach, the recently developed VasCog Screen test showed good sensitivity, specificity, and classification accuracy for detecting cognitive deficits in patients after stroke and patients with HF [248]. Previous studies have mainly emphasized the roles of reduced cardiac output, arterial stiffness, and chronic cerebral hypoperfusion in CI. Recent evidence suggests that ventricular structural characteristics, including myocardial mass, wall thickness, and ventricular volume, constitute the cardiovascular phenotype most strongly associated with hippocampal and subcortical brain volumes [249]. In contrast, left ventricular systolic function, diastolic function, and aortic compliance show weaker associations with brain structure and no association with cognitive performance [249]. These findings suggest that ventricular structural phenotypes may provide a new basis for early screening and risk assessment.

The comprehensive management of patients with coexisting CI and cardiac disease requires a multidisciplinary approach. Interventions can be broadly categorized into three domains: (1) risk factor optimization, (2) nonpharmacological interventions, and (3) pharmacological therapy [250].

Comprehensive management of cardiovascular risk factors is central to improving cognitive outcomes. This includes adhering to lifestyle recommendations such as “Life's Essential 8,” controlling BP, managing blood glucose with preferential use of agents that may have heart–brain benefits, such as GLP‐1 receptor agonists, and the appropriate use of statins. Consistent with this approach, a prospective study showed that the SGLT2 inhibitor empagliflozin was associated with improved cognitive function in older patients with diabetes and HFpEF [251]. Furthermore, guideline‐directed treatment of specific cardiac conditions, including AF, HF, and severe aortic stenosis, may contribute to the preservation of cognitive function. Clinical management should therefore incorporate individualized strategies that balance potential benefits and risks based on patient‐specific factors [250]. For instance, a multicenter study found that ACE inhibitor use in patients with HF was associated with cognitive function (OR = 1.57) [252]. Similarly, catheter ablation for rhythm control in AF may improve cognitive performance, particularly in the domains of visuospatial ability, short‐term memory, attention, and language [253]. Moreover, a large Swedish retrospective study showed that guideline‐adherent anticoagulation was associated with a 29% lower risk of dementia in patients with AF [222]. Regarding device therapy, cardiac resynchronization therapy was associated with improved cognitive performance in a study of 45 patients with HF and severe left ventricular dysfunction [254].

Nonpharmacological interventions are supported as strategies for delaying cognitive decline. These interventions include regular physical exercise, particularly resistance training, healthy dietary patterns, smoking cessation, management of sleep disorders, and increased social engagement, all of which may provide cognitive benefits [250]. A multidimensional approach integrating exercise, cognitive training, and vascular risk monitoring may further reduce the risk of cognitive decline in high‐risk populations, highlighting the potential benefits of comprehensive management [250]. For instance, a small randomized controlled trial reported that light‐to‐moderate exercise improved cognitive function in patients with HF [255]. However, a larger multicenter randomized controlled trial found no significant improvement in cognitive function with a multimodal physical rehabilitation program in patients with HF [256]. Thus, the precise impact of exercise training on cognition in HF requires further evaluation.

For CI, cholinesterase inhibitors and memantine may provide short‐term symptomatic benefits but require monitoring for potential adverse cardiovascular effects. Novel anti‐Aβ monoclonal antibodies, such as lecanemab and donanemab, may slow disease progression but are associated with risks of cerebral edema and hemorrhage and have limited applicability. In contrast, antiplatelet agents have not demonstrated efficacy in the primary prevention of cognitive decline. Therefore, pharmacological management requires a rigorous benefit‐risk assessment, particularly in patients with coexisting CVD [250].

**FIGURE 5:**
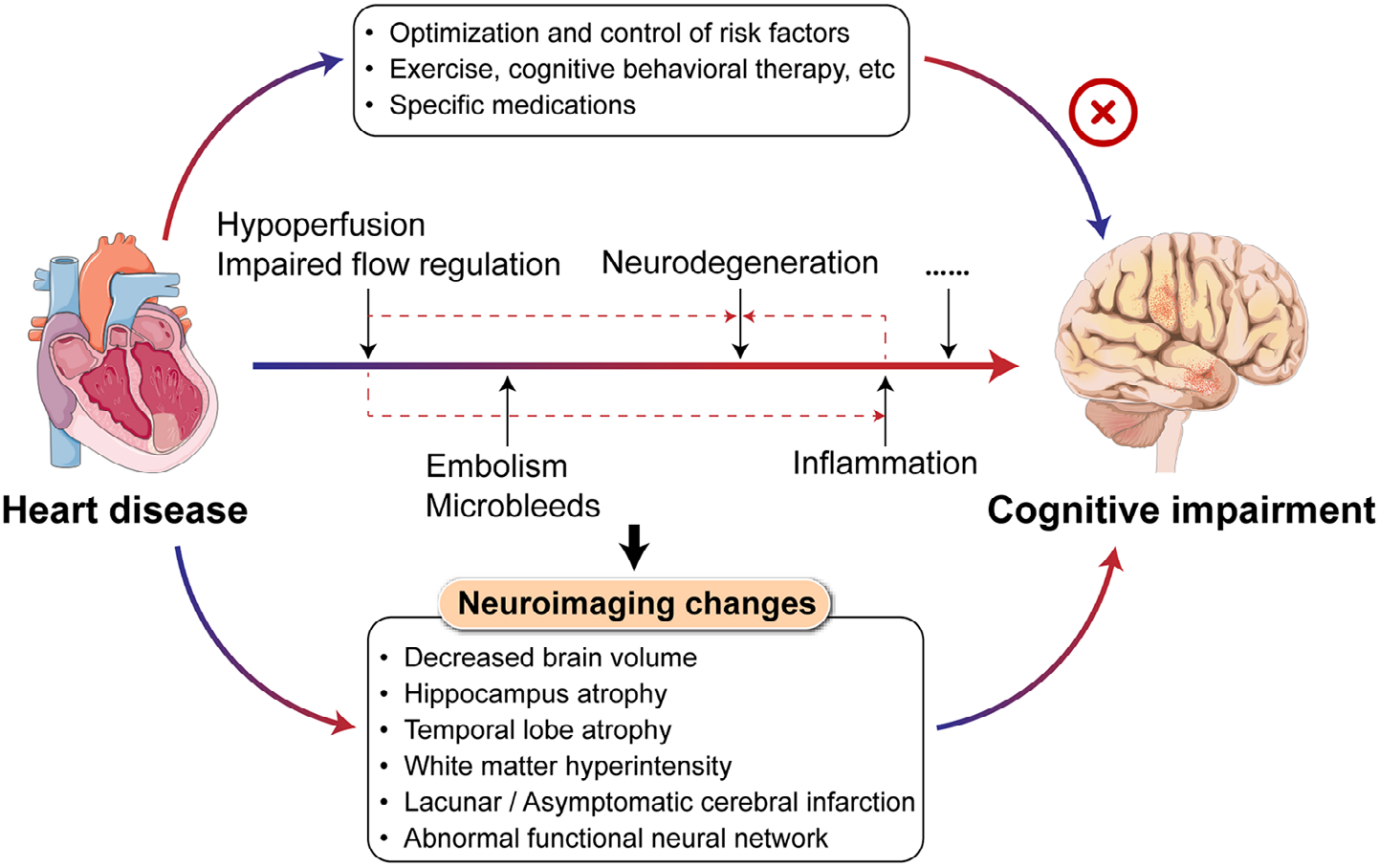
Heart–brain interactions in cardiac dementia. Cardiac diseases may contribute to cognitive impairment through cerebral hypoperfusion, impaired cerebral blood flow regulation, cerebral embolic events, cerebral microbleeds, inflammation, and neurodegeneration. These mechanisms are associated with neuroimaging abnormalities, including reduced brain volume, hippocampal and temporal lobe atrophy, white matter hyperintensities, lacunar infarcts and silent cerebral infarcts, and abnormal functional brain networks. Integrated management may include cardiovascular risk factor control, exercise interventions and cognitive behavioral training, and disease‐specific pharmacological therapies to help delay cognitive decline in patients with cardiac disease.

Current research on CI in patients with cardiac disease predominantly focuses on clinical phenotypes, whereas the underlying mechanisms remain insufficiently understood (Figure 5). Future studies should therefore prioritize elucidating the pathophysiological mechanisms of cardiac dementia, with key directions including: (1) developing pathogenesis‐based targeted interventions; (2) establishing a multimodal biomarker system that integrates cardiac and neurodegenerative profiles to guide precision therapy; and (3) investigating the neuroprotective mechanisms of nonpharmacological interventions. Integrated heart–brain management represents a promising approach to slowing the progression of CI in this vulnerable population.

### 3.6 Psychocardiology

Psychocardiology is an interdisciplinary field examining the bidirectional interplay between psychological factors and CVD. A core tenet of psychocardiology is that psychological disorders, particularly depression, are independent risk factors for the development of CVD and poor cardiovascular outcomes, which may, in turn, contribute to the development or exacerbation of psychological conditions. This psychosomatic interplay may establish a vicious cycle, resulting in adverse clinical outcomes [257, 258]. Consequently, the field advocates an integrated management model incorporating psychological screening and care into cardiovascular management to interrupt this cycle and improve both psychological and cardiovascular outcomes [257, 258]. Treatment also requires careful consideration of the potential cardiovascular toxicity of psychotropic agents and their pharmacokinetic interactions with cardiovascular medications [259].

#### 3.6.1 Epidemiology of Psychocardiological Conditions

Substantial epidemiological evidence supports a strong association between psychological factors and CVD. For instance, a large multinational cohort study showed that depressive symptoms (≥4) were associated with increased risks of cardiovascular events (HR = 1.14) and all‐cause mortality (HR = 1.17), with the risks increasing with greater symptom burden. This association was especially pronounced in urban residents and males [260]. A meta‐analysis of 37 prospective studies showed that anxiety disorders were associated with a 52% higher incidence of CVD [261]. Similarly, a study of 138,000 older veterans linked posttraumatic stress disorder to an elevated risk of cerebrovascular disease, MI, and HF [262]. Beyond specific disorders, adverse psychosocial factors, such as social isolation, financial strain, and work stress, are associated with elevated cardiovascular risk. In contrast, positive psychological traits, such as optimism and life satisfaction, are associated with lower cardiovascular risk [263].

CVD, in turn, can adversely affect mental health. The threat of death from acute events and the long‐term burden of chronic disease may contribute to depression, anxiety, and posttraumatic stress disorder. These psychological comorbidities can substantially reduce quality of life and impair treatment adherence and self‐management capacity [263]. A systematic review of 68 studies reported high prevalence of depression (31.3%), anxiety (32.9%), and stress‐related disorders (57.7%) among patients with CVD [264]. Furthermore, heart disease may contribute to persistent negative psychological states [263].

**FIGURE 6:**
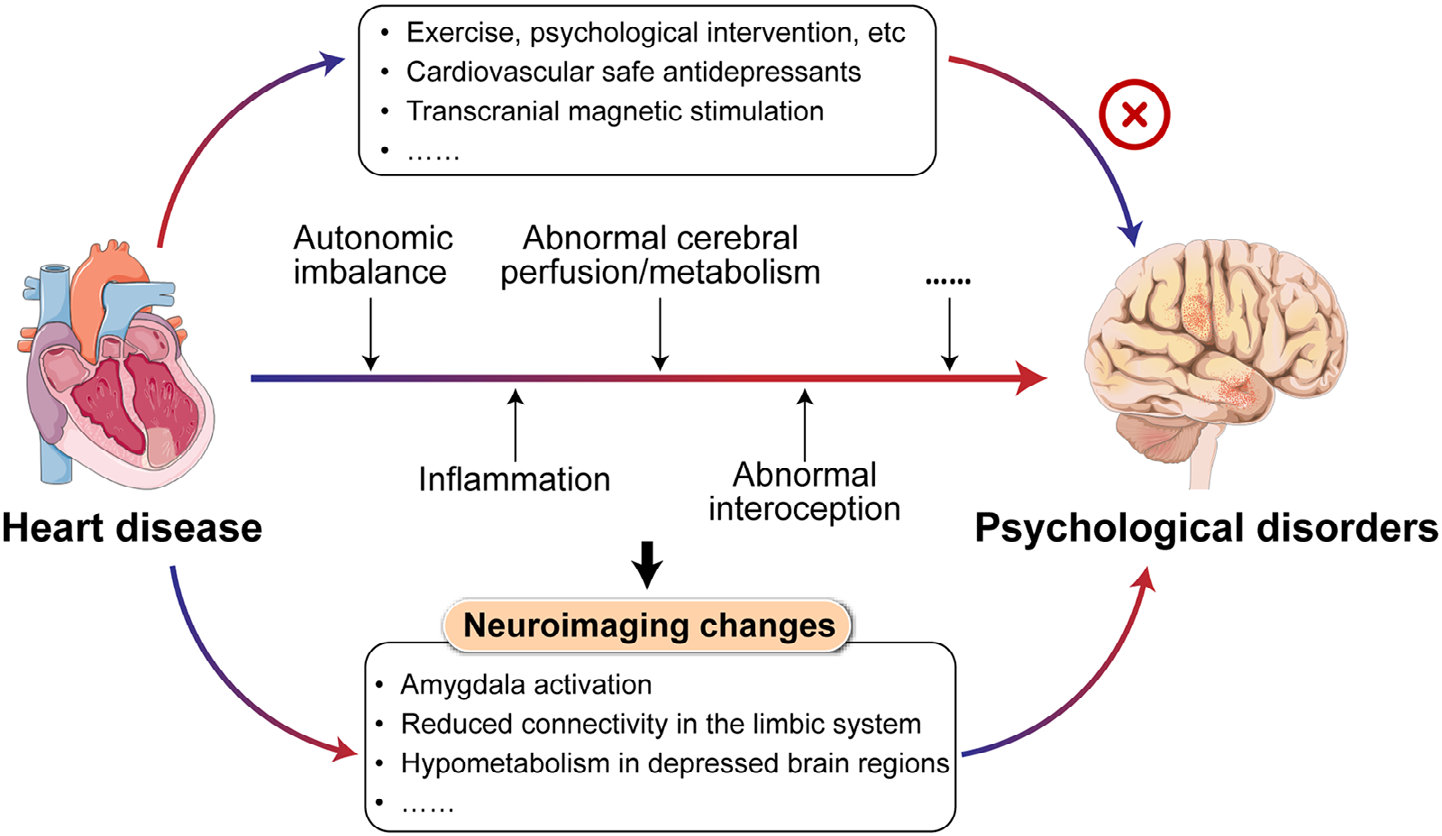
Heart–brain interactions in psychocardiology. Cardiac diseases may contribute to psychological symptoms and disorders through autonomic imbalance, inflammation, abnormalities in cerebral perfusion or metabolism, and altered interoception. These mechanisms are associated with neuroimaging abnormalities, including amygdala hyperactivity, reduced functional connectivity within limbic networks, and regional hypometabolism in depression‐related brain areas. Integrated management may include exercise training, psychological interventions, antidepressants with favorable cardiovascular safety profiles, transcranial magnetic stimulation, and other individualized treatment strategies to alleviate psychological distress and potentially improve cardiovascular outcomes.

These psychological conditions are associated with worse clinical outcomes. Depression is associated with a 40% higher risk of all‐cause mortality among patients with HF [264]. Among patients with HFrEF, depression is associated with higher risk of all‐cause hospitalization (HR = 1.42), cardiovascular death (HR = 1.69), and all‐cause mortality (HR = 1.54) [265]. Similarly, generalized anxiety disorder is associated with an approximately twofold higher risk of adverse outcomes in patients with MI [266], and dissociative symptoms are independently associated with 15‐year all‐cause mortality in this population [267]. The association between psychological disorders and poor treatment adherence is well documented. For example, an analysis of 124,000 patients after percutaneous coronary intervention showed that patients with depression (16.6%) had lower adherence to guideline‐directed medical therapy [268]. These findings collectively support a bidirectional relationship between CVD and mental health, potentially creating a self‐perpetuating cycle (Figure 6).

#### 3.6.2 Pathophysiological Mechanisms of Psychocardiology

##### 3.6.2.1 Autonomic Dysregulation

The core pathophysiological mechanisms underlying psychocardiological conditions involve persistent dysregulation of the ANS and the HPA axis [257, 258]. Patients with depression frequently exhibit cardiac autonomic imbalance, characterized by reduced high‐frequency power, increased low‐frequency power, and an elevated low‐frequency‐to‐high‐frequency ratio—a pattern indicative of suppressed parasympathetic activity and concomitant sympathetic overactivation [269]. The severity of this autonomic dysfunction shows a positive linear correlation with depression severity [269]. A cross‐sectional study found an inverse correlation between depression scores and HRV parameters [270]. Furthermore, a model based on resting cardiac autonomic balance showed potential for identifying major depressive disorder (OR = 0.70) [271]. At the molecular level, animal studies indicate that sigma‐1 receptor stimulation may ameliorate depression‐related autonomic dysfunction. This effect may be mediated by the inhibition of L‐type calcium currents and restoration of transient outward potassium currents, thereby reducing susceptibility to arrhythmias [272, 273].

Studies reveal distinct sex‐specific pathophysiological characteristics in depression: men with depression exhibit reduced HRV during passive tilt‐table testing, whereas women with depression show no clear change in autonomic function [274]. In contrast, women with depression exhibit maladaptive CNS alterations, including hyperactivity of the hypothalamus and right amygdala, alongside reduced hypothalamic connectivity with the right orbitofrontal cortex, amygdala, and hippocampus [275]. These findings suggest sex‐dependent associations between depression and cardiac autonomic dysfunction.

Furthermore, depression may induce sympathetic nerve‐mediated immune dysregulation. In patients with HF and depressive symptoms, peripheral blood mononuclear cells exhibit an enhanced chemotactic response to isoproterenol after acute exercise, suggesting increased β‐adrenergic receptor sensitivity [276]. This sympathetic overactivation may consequently contribute to myocardial remodeling by promoting immune cell migration [276].

##### 3.6.2.2 Inflammation

Inflammation may represent a key mechanism linking depression and cardiac arrhythmia. Clinically, patients with major depressive disorder exhibit elevated plasma concentrations of 18 pro‐ and anti‐inflammatory cytokines, consistent with systemic inflammation [277]. Animal studies further suggest that depression may activate the NLRP3 inflammasome through a P2×7 receptor‐dependent pathway, thereby contributing to AF [278]. Notably, P2×7 receptor inhibition not only ameliorated autonomic dysfunction but also reduced atrial fibrosis and normalized ion channel expression, thereby alleviating depression‐like behavior and reducing the occurrence of AF [278].

In CHF, major depressive disorder is associated with elevated levels of inflammatory markers, including IL‐6, IL‐8, and interferon‐γ [279]. A retrospective study developed a predictive model for depression severity that integrated cardiac function parameters with inflammatory markers, with IL‐6 and TNF‐α contributing to the model's predictive performance [280]. At the mechanistic level, HF induces a cerebral inflammatory cascade in mouse models. This process activates the tryptophan–kynurenine pathway, leading to accumulation of neurotoxic metabolites, which subsequently disrupts glutamatergic balance and synaptic plasticity [281]. In parallel, HF activates the HPA axis, which further exacerbates neuroinflammation and oxidative stress, ultimately contributing to depression‐like behaviors [281]. Collectively, these findings support an important role for inflammatory mechanisms in the association between HF and depression, highlighting their relevance to psychocardiology.

##### 3.6.2.3 Interoceptive Dysfunction

Abnormal interoception may represent an important pathophysiological basis for psychiatric disorders, including depression and anxiety [282]. Using optogenetics, Hsueh et al. demonstrated that artificially induced tachycardia in a threatening context enhanced anxiety‐like behaviors in mice, demonstrating that peripheral cardiac signals can directly modulate emotional states [17]. Further mechanistic experiments showed that optogenetic inhibition of the posterior insular cortex, a key hub for cardiac interoception, attenuated this pacing‐induced anxiety‐like behavior [17].

These findings suggest a potential mechanistic explanation for the high prevalence of depression in patients with AF: AF‐related rhythm abnormalities may transmit aberrant interoceptive signals to the CNS through a bottom‐up pathway, predominantly disrupting posterior insular cortex function and thereby contributing to emotional dysregulation. This proposed mechanism suggests a specific pathophysiological pathway from aberrant cardiac activity to central emotional dysregulation.

##### 3.6.2.4 Cerebral Perfusion and Metabolic Abnormalities

HF‐related cerebral hypoperfusion may lead to characteristic alterations in brain metabolism. ^1^ ^8^F‐FDG PET/CT studies have shown that global brain metabolism in patients with HFrEF is correlated with NYHA functional class, depression severity, and CI. Key emotion‐regulating regions, including the ventral prefrontal cortex, amygdala, and hippocampus, exhibit a specific pattern of hypometabolism [283]. Notably, a standardized metabolic ratio (depression‐related regions‐to‐whole‐brain) ≤0.93 differentiates primary major depression from secondary “pseudo‐depression”: globally reduced metabolism may suggest a secondary condition, whereas region‐specific hypometabolism may be more consistent with primary major depression [283]. Furthermore, common HF comorbidities, including AF, stroke, and sleep‐disordered breathing, may independently affect these key emotion‐regulating regions, thereby contributing to cognitive dysfunction and an increased risk of depression [284].

At the myocardial metabolic level, mice with chronic stress‐induced depression exhibit alterations in cardiac glycosaminoglycan profile, notably elevated levels of heparin and chondroitin sulfate. These alterations may promote myocardial hypertrophy through BDNF–TrkB signaling and inflammatory pathways [285]. Similarly, a rat model of depression showed myocardial metabolic remodeling, featuring reduced NAD^+^ and NADP^+^ levels and increased NADPH levels, disordered amino acid metabolism, and increased DNA damage [286]. Histopathological examination further showed that depression was associated with structural myocardial changes, including increased cardiomyocyte eosinophilia, perivascular collagen deposition, and abnormal glycogen accumulation [286]. Collectively, these findings support metabolic heart–brain interactions that may link psychological stress to structural cardiac pathology.

#### 3.6.3 Integrated Heart–Brain Management of Psychocardiology

The 2025 ESC Clinical Consensus Statement on Mental Health and Cardiovascular Diseases introduced the “Psycho–Cardio Team” concept, highlighting the important role of multidisciplinary collaboration in managing patients with coexisting mental health and cardiovascular conditions. The statement recommends the ACTIVE principle as a practical framework for integrating mental health care into cardiovascular management. This framework comprises six components: acknowledge, check, tools, implement, venture, and evaluate [263].

Mental health screening should be routinely implemented in patients with CVD. Initial screening can be conducted using Whooley questions, PHQ‐2, or GAD‐2, with positive screening results warranting further assessment using PHQ‐9, GAD‐7, or HADS. Screening is recommended at the time of new diagnosis, during annual follow‐up visits, and when clinical indicated. A stepped‐care approach is recommended: Level 1 entails universal health education and general support; Level 2 provides structured psychotherapy for mild‐to‐moderate symptoms; and Level 3 involves pharmacological treatment and referral to a specialist for moderate‐to‐severe symptoms. This approach provides a standardized closed‐loop care pathway encompassing screening, assessment, and intervention [263].

Available nonpharmacological interventions include psychological therapies, such as CBT, exposure therapy, and behavioral activation, psychoeducation, social prescribing, lifestyle modifications, including physical activity, structured exercise, dietary interventions, and smoking cessation, stress management techniques, such as meditation, mindfulness, and progressive muscle relaxation, and optimization of sleep hygiene. Supportive approaches, including digital health tools, motivational interviewing, and comprehensive cardiac rehabilitation programs, may facilitate the implementation and maintenance of lifestyle modifications [263].

Key nonpharmacological approaches for managing depression in patients with cardiac disease include exercise training, CBT, comprehensive cardiac rehabilitation, and palliative care [263]. In patients with paroxysmal AF, yoga training reduced symptomatic episode frequency by 45% (from 3.8 ± 3 to 2.1 ± 2.6 episodes) and was also associated with lower anxiety and depression scores, improved hemodynamic parameters, and better quality of life [287]. The HF‐ACTION trial showed that aerobic exercise reduced depressive symptoms in patients with HFrEF at both the 3‐ and 12‐month follow‐ups [288]. While both aerobic exercise and cardiac rehabilitation improve cardiovascular health and depressive symptoms, exercise prescriptions should be individualized according to the patient's cardiac status and exercise capacity. Regarding psychological interventions, a randomized controlled trial found that CBT improved health‐related quality of life, illness perception, and depressive symptoms in patients with AF [289]. Another RCT demonstrated that CBT reduced depression, anxiety, fatigue and improved social functioning and health‐related quality of life in patients with HF and major depressive disorder, though it showed no significant effects on HF self‐care or physical function [290]. A recent meta‐analysis found that CBT was the only intervention associated with a significant reduction in depression scores, whereas the effects of other interventions were not significant [291], suggesting that CBT may be a preferred psychological treatment for patients with HF and coexisting depression. The PAL‐HF trial showed that palliative care improved quality of life and reduced anxiety and depression in patients with advanced HF [292]. However, ESC guidelines note that no consensus exists regarding the optimal treatment approach for patients with HF and depression [4]. A recent RCT showed that both behavioral activation psychotherapy and antidepressant medication were associated with nearly 50% reductions in depressive symptoms over 12 months, with no significant difference between the two approaches [293]. Collectively, these findings support integrating psychological interventions and exercise therapy into management plans for patients with cardiac disease, although their long‐term benefits require further evaluation in larger trials.

For patients with severe mental health conditions, pharmacotherapy may be indicated, with the choice of antidepressants, anxiolytics, sedative‐hypnotics, mood stabilizers, or antipsychotics based on the specific symptom profile and severity [263]. According to ESC guidelines, psychosocial interventions and selective serotonin reuptake inhibitors may improve depressive symptoms in patients with HF but have not been shown to improve clinical outcomes [4]. Sertraline and escitalopram are generally considered to have favorable cardiovascular safety profiles, whereas tricyclic antidepressants are generally avoided because of the risks of hypotension, HF exacerbation, and arrhythmias [4]. A large Danish cohort study showed a 3.18‐fold higher risk of AF (aHR = 3.18) during the first month of antidepressant treatment, with this association attenuating over time (aHR = 1.37 at 2–6 months; aHR = 1.11 at 6–12 months) [294]. Notably, the risk of AF was already elevated during the 15–30 days before treatment initiation (aHR = 7.65) [294]. Although antidepressant therapy may provide an overall net benefit, cardiovascular adverse effects may occur and may be obscured by therapeutic benefits. A meta‐analysis showed that patients with HF and depression who received antidepressants had higher risks of all‐cause and cardiovascular mortality [295]. Consequently, careful monitoring of cardiovascular effects is warranted, as some antidepressants may suppress cardiac vagal tone and potentially contribute to arrhythmias, autonomic dysfunction, endocrine disturbances, and sleep disorders [259]. Further development of antidepressants with improved cardiovascular safety profiles is needed.

Transcranial magnetic stimulation offers several advantages for treating depression, including its noninvasive nature, absence of systemic drug side effects, efficacy in treatment‐resistant depression, targeted neuromodulation capability, and favorable safety profile without the need for general anesthesia. While transcranial magnetic stimulation represents a promising nonpharmacological intervention for depression in patients with HF, its use requires careful safety assessment in patients with pacemakers or other cardiac implantable electronic devices. Regarding nutritional interventions, the OCEAN trial suggested that omega‐3 fatty acid supplementation may improve depressive symptoms in patients with CHF and major depressive disorder [296]; however, these findings require further evaluation in larger trials.

## 4 Clinical Trials Targeting Heart–Brain Interactions

Current clinical research on diseases involving heart–brain interactions is gradually shifting from single‐organ treatment toward integrated heart–brain management. Based on the aims and intervention strategies of clinical trials across the six disease categories, these studies can be broadly classified into six categories: autonomic modulation, biomarker monitoring, neuroimaging assessment, exercise therapy, psychological interventions, and pharmacological therapy. Detailed information on these clinical trials is summarized in Table 1.

**TABLE 1:** Clinical trials targeting heart–brain interactions.

Category	Trial	Registration no.	Disease context	Status/phase	Intervention/research focus	Key findings
Autonomic modulation	Autonomic modulation in takotsubo syndrome/Tako‐breathe	NCT03324529	Takotsubo syndrome	Active, not recruiting; N/A	Breathing‐based autonomic modulation	Not available
	Sympathetic heart innervation in patients with takotsubo cardiomyopathy	NCT01524861	Takotsubo syndrome	Completed; Phase 4	α‐Lipoic acid plus acetyl‐L‐carnitine for sympathetic modulation	Not available
	Sympathetic and vascular function in Takotsubo syndrome/SAFT	NCT05768542	Takotsubo syndrome	Completed; Phase 2	Sympathetic activity, vascular function, and metoprolol response	Resting and stress‐induced norepinephrine levels, as well as muscle sympathetic nerve activity, were comparable between patients with TTS and controls [297].
	β‐Tako trial	NCT06509074	Takotsubo syndrome	Not yet recruiting; Phase 4	β‐Blocker therapy; sympathetic modulation	Not available
	Seizure detection and automatic magnet mode performance study (E‐36)	NCT01325623	Epileptic heart	Completed; N/A	Heart rate‐triggered VNS	VNS may reduce indices of cardiac electrical instability [298].
	VNS therapy automatic magnet mode outcomes study in epilepsy patients exhibiting ictal tachycardia (E‐37)	NCT01846741	Epileptic heart	Completed; N/A	Heart rate‐triggered VNS	VNS was well tolerated and was associated with improved clinical outcomes [299].
	Transcutaneous auricular VNS for pediatric epilepsy	NCT02004340	Epileptic heart	Unknown status; N/A	Transcutaneous auricular VNS	Seizure frequency was reduced by approximately 54.13% [300].
	ta‐VNS for drug‐resistant epilepsy	N/A	Epileptic heart	N/A	Transcutaneous auricular VNS	The mean seizure frequency decreased by 30.75% [301].
	Propranolol adjuvant treatment of focal refractory epilepsy	NCT06719804	Epileptic heart	Not yet recruiting; early Phase 1	Propranolol as adjunctive therapy for drug‐resistant focal epilepsy	Not available
	Impact of remote ischemic postconditioning on autonomic function in stroke patients/IRAS	NCT02777099	Stroke–heart syndrome	Unknown status; N/A	Remote ischemic postconditioning; assessment of HRV and autonomic function	Remote ischemic postconditioning improved neurological outcomes after AIS; changes in autonomic function may mediate this effect [302].
	The effect of endovascular thrombectomy on autonomic nervous system in large‐vessel ischemic stroke/AFFRICATE	NCT05099354	Stroke–heart syndrome	Unknown status; N/A	Endovascular thrombectomy; autonomic nervous system; HRV and baroreflex sensitivity	Not available
	Evaluation of autonomic modulation in stroke after tDCS and treadmill training	NCT02956096	Stroke–heart syndrome	Unknown status; N/A	Transcranial direct current stimulation and treadmill training; assessment of HRV and blood pressure variability	No immediate effect on HRV or systolic blood pressure variability were observed; left dorsolateral prefrontal stimulation may improve cardiac autonomic responses [303].
	Neural cardiac therapy for heart failure/NECTAR‐HF	NCT01385176	Heart failure	Active, not recruiting; N/A	VNS in HFrEF; assessment of left ventricular remodeling, exercise capacity, and QoL	VNS showed favorable long‐term safety but did not improve efficacy endpoints [304, 305].
	Autonomic neural regulation therapy to enhance myocardial function in heart failure/ANTHEM‐HF	NCT01823887	Heart failure	Completed; Phase 2	VNS for autonomic modulation in HFrEF	Sustained autonomic engagement was observed during up to 5 years of follow‐up with autonomic regulation therapy [306].
	ANTHEM‐HFrEF pivotal study	NCT03425422	Heart failure	Terminated; N/A	VITARIA system‐based VNS in HFrEF	VNS may improve symptoms and cardiac function and may be associated with better clinical outcomes [307].
	ANTHEM‐HFpEF study	NCT03163030	Heart failure	Completed; N/A	VNS in patients with HFpEF	Not available
	Low‐level transcutaneous vagus nerve stimulation in HFpEF	NCT03327649	Heart failure	Completed; N/A	Low‐level transcutaneous VNS in HFpEF; assessment of inflammatory markers	Low‐level transcutaneous VNS improved global longitudinal strain and QoL and reduced inflammatory cytokine levels in patients with HFpEF [194].
	TRAnscutaneous vaGUS nerve stimulation in patients with chronic heart failure (TRAGUS‐HF)	NCT06355388	Heart failure	Not yet recruiting; N/A	Transcutaneous VNS in chronic heart failure	Acute right‐sided tVNS increased cardiovagal baroreflex gain without major tolerability concerns [308].
	Low‐level tragus stimulation in acute decompensated heart failure/TREAT‐HF	NCT02898181	Heart failure	Recruiting; N/A	Low‐level tragus stimulation in acute decompensated HF; assessment of inflammation and autonomic modulation	Low‐level tragus stimulation attenuated systemic inflammation and coronary endothelial oxidative stress [309].
	BAROSTIM hope for heart failure/HOPE4HF	NCT01720160	Heart failure	Completed; N/A	BAT in patients with HFrEF	BAT improved QoL, exercise capacity, NT‐proBNP levels, and LVEF and reduced HF hospitalizations, particularly among patients not receiving cardiac resynchronization therapy [310].
	Baroreflex activation therapy for heart failure/BeAT‐HF	NCT02627196	Heart failure	Completed; N/A	BAT in patients with HFrEF	BAT improved physical and emotional scores in patients with HFrEF [311].
	Renal denervation in chronic heart failure	NCT02146794	Heart failure	Completed; Phase 1	Renal sympathetic denervation in chronic heart failure	Not available
	Spinal cord stimulation for heart failure/SCS HEART	NCT01362725	Heart failure	Completed; Phase 3	SCS in patients with severe symptomatic HF	SCS was safe and feasible and improved symptoms, functional status, LV function, and cardiac remodeling in patients with systolic HF [312].
	Spinal cord stimulation in heart failure	NCT03060148	Heart failure	Unknown status; N/A	SCS in patients with severe symptomatic HF	Not available
	Surgical resection of the greater splanchnic nerve in HFpEF	NCT03715543	Heart failure	Completed; N/A	Right GSN resection in patients with HFpEF	Right GSN resection improved exercise‐induced volume redistribution and reduced elevated filling pressures in patients with HFpEF [313].
	Endovascular GSN ablation in subjects with HFpEF	NCT04287946	Heart failure	Unknown status; N/A	Right GSN block or ablation in patients with HFpEF	Right GSN block or ablation was associated with improved cardiac function in patients with HFpEF [199].
	Endovascular ablation of the right greater splanchnic nerve in HFpEF/REBALANCE‐HF	NCT04592445	Heart failure	Recruiting; N/A	Endovascular ablation of the right GSN in patients with HFpEF	Endovascular ablation of the right GSN did not improve exercise PCWP or clinical status in patients with HFpEF [314].
	MOXCON trial	N/A	Heart failure	Terminated; N/A	Central sympathetic inhibition with moxonidine in chronic heart failure	Moxonidine reduced norepinephrine levels but increased early mortality and adverse events [192].
Biomarker and electrophysiological monitoring	Cardiac arrhythmias in Dravet syndrome	NCT02415686	Epileptic heart	Completed; N/A	Long‐term ECG monitoring in patients with Dravet syndrome	Ictal QTc prolongation was more frequent in patients with Dravet syndrome [315].
	Heart rate variability analysis in patients with epilepsy	NCT02469844	Epileptic heart	Completed; N/A	Preictal HRV analysis during sleep	Not available
	Heart rate changes in subjects with epilepsy	NCT01202669	Epileptic heart	Completed; N/A	Continuous heart rate monitoring in the epilepsy monitoring unit	Not available
	Heart rate changes during normal activity, exercise, and epileptic seizures	NCT01214707	Epileptic heart	Completed; N/A	Comparison of ECG and heart rate changes during daily activities and exercise and before and after epileptic seizures	Not available
	Physiological and biomechanical data collection study in epilepsy	NCT01485016	Epileptic heart	Completed; N/A	Wearable ECG; respiratory, and physiological monitoring	Not available
	Seizure alarm with wearable ECG device	ISRCTN85640482	Epileptic heart	Completed; Phase 3	Wearable ECG patch with a real‐time smartphone‐based algorithm	The wearable ECG device accurately detected focal and generalized motor seizures [127].
	Heart‐rate‐variability‐based seizure detection using wearable ECG	N/A	Epileptic heart	N/A	Wearable ECG with an HRV‐based seizure detection algorithm	In patients whose seizures were accompanied by marked autonomic changes, the algorithm achieved a sensitivity of 87.0% [316].
	Development of a minimally invasive seizure gauge	NCT03882671	Epileptic heart	Completed; N/A	Combined EEG and ECG monitoring	Not available
	Mechanisms and prognosis of stroke–heart syndrome/MAP‐SHS	NCT06954103	Stroke–heart syndrome	Recruiting; N/A	Investigation of SHS mechanisms using multimodal imaging, blood‐based multiomics, and HRV	Not available
	Cardiomyocyte injury following acute ischemic stroke/CORONA‐IS	NCT03892226	Stroke–heart syndrome	Unknown status; N/A	hs‐cTn, CMR, echocardiography, and autonomic ECG markers	Not available
	Imaging and serological biomarkers of autonomic dysfunction after ischemic stroke/ARIADNE	NCT06740942	Stroke–heart syndrome	Not yet recruiting; N/A	Autonomic dysfunction after AIS; CAN; imaging and serological biomarkers	Not available
	Stroke arrhythmia monitoring database/SAMBA	NCT01177748	Stroke–heart syndrome	Completed; N/A	Arrhythmia monitoring after acute stroke using continuous ECG and telemetry	The risk of severe arrhythmias was highest within 24 h after acute cerebrovascular events and declined over the subsequent 3 days [143].
	Effects of heart rate variability biofeedback in patients with acute ischemic stroke/Strokeback01	NCT03865225	Stroke–heart syndrome	Completed; N/A	HRV biofeedback for autonomic modulation after AIS	HRV biofeedback improved cardiac autonomic function when added to standard stroke care [317].
Neuroimaging	Brain fMRI in Takotsubo cardiomyopathy	NCT02240056	Takotsubo syndrome	Completed; N/A	Brain fMRI	Acute TTS was associated with alterations in the temporal lobe and CAN, including structural and functional changes in the right insula [86].
	Brain–heart Interactions in Takotsubo cardiomyopathy and cardiac syndrome X	NCT02759341	Takotsubo syndrome	Completed; N/A	Brain imaging and assessment of neuropsychological–cardiovascular interactions	Patients with TTS showed increased functional integration within CAN regions involved in pain and negative emotions [98].
	TRAnscranial Doppler CErebral blood flow and cognitive impairment/TRACE‐IMPAIR	NCT05250349	Cardiac dementia	Recruiting; N/A	Assessment of cerebral blood flow and cognitive impairment in HF using transcranial Doppler	Not available
	Cardiovascular risk factors and cognitive trajectories	NCT05441865	Cardiac dementia	Completed; N/A	Assessment of cardiovascular risk factors, cerebral blood flow, brain functional connectivity, and cognitive trajectories	Not available
Exercise and lifestyle interventions	BREAKOUT study: lifestyle interventions for modulating the brain phenotype of Takotsubo cardiomyopathy	NCT05530135	Takotsubo syndrome	Completed; N/A	CBT and exercise training; assessment using brain imaging	A 12‐week behavioral intervention was associated with improvements in brain activity, brain structure, inflammatory markers, and psychological well‐being after TTS [110].
	PLEASE study	NCT04425785	Takotsubo syndrome	Completed; N/A	Exercise training and CBT	Exercise training or CBT improved myocardial energetics and exercise capacity in patients with TTS [318].
	Randomized controlled trial on cardiovascular exercise in epilepsy	NCT03570489	Epileptic heart	Terminated; N/A	Cardiovascular exercise training	Cardiovascular exercise training did not reduce seizure frequency in patients with drug‐resistant epilepsy [319].
	Interval versus continuous training in heart failure	NCT02448147	Heart failure	Completed; N/A	Comparison of interval and continuous training; assessment of sympathetic activity and peripheral muscle perfusion	Interval training improved skeletal muscle oxygenation and blood flow and reduced sympathetic nerve activity in patients with HFrEF [320].
	Impact of interval training on sympathetic hyperactivity in HF	NCT04248894	Heart failure	Completed; N/A	HIIT; assessment of sympathetic activity	HIIT reduced muscle sympathetic nerve activity and improved peripheral vascular function in patients with HFrEF [321].
	Exercise and cognitive retraining to improve cognition in heart failure	NCT02151266	Cardiac dementia	Completed; N/A	Exercise training combined with cognitive training in patients with HF‐related cognitive impairment	Aerobic exercise combined with cognitive training improved memory in patients with HF [322].
	ENLIGHTEN: exercise and diet intervention for cognitive function in CVD Risk	NCT01573546	Cardiac dementia	Completed; N/A	Exercise and diet intervention for cognitive function in individuals at risk of CVD	Aerobic exercise combined with the DASH diet improved executive function in individuals at cardiovascular risk [323].
Psychological interventions	Psychosocial support for patients with Takotsubo syndrome	NCT05977049	Takotsubo syndrome	Recruiting; N/A	Psychological support and anxiety management through a Takotsubo support group	Not available
	E‐SMINC: Internet‐based treatment of stress and anxiety in MINOCA or Takotsubo syndrome	NCT04178434	Takotsubo syndrome	Recruiting; N/A	Internet‐based CBT	Internet‐based CBT improved psychological well‐being and QoL [324].
	Computerized cognitive training in patients with coronary heart disease	NCT05735041	Cardiac dementia	Unknown status; N/A	CCT in patients with coronary heart disease	Adaptive CCT improved adherence but did not outperform traditional CCT in terms of cognitive outcomes [325].
	Internet‐based behavioral intervention following ACS	NCT06298864	Psychocardiology	Recruiting; N/A	Internet‐based CBT for anxiety after ACS	Not available
	Brief Internet‐delivered CBT After ACS	NCT05607992	Psychocardiology	Completed; N/A	Brief internet‐delivered exposure‐based CBT for anxiety after ACS	Not available
	Stepped care for depression in heart failure	NCT02997865	Psychocardiology	Completed; N/A	Stepped‐care CBT for patients with HF and depression	Stepped‐care CBT improved depressive symptoms and QoL compared with usual care, with benefits sustained for at least 1 year [326].
	Blended collaborative care for heart failure and comorbid depression	NCT02044211	Psychocardiology	Completed; Phase 3	Telephone‐based collaborative care for patients with HF and depression	Telephone‐based collaborative care did not reduce readmission or mortality but resulted in greater improvement in mood [327].
	Personalized treatments for depressive symptoms in advanced heart failure	NCT03688100	Psychocardiology	Completed; Phase 4	Behavioral activation psychotherapy versus antidepressant medication in patients with advanced HF and depression	Both treatments reduced depressive symptoms by nearly 50%, with no significant between‐group difference [293].
	Efficacy of CBT on positive health outcomes of chronic heart failure patients with depression	NCT04949945	Psychocardiology	Unknown status; N/A	Culturally adapted CBT for patients with chronic HF and depression	Not available
	Atrial fibrillation, cardiac symptoms, and anxiety	NCT02133365	Psychocardiology	Completed; N/A	Mindfulness and interoceptive exposure for anxiety and AF‐related symptoms	Not available
	Effects of mindfulness on atrial fibrillation	NCT02564302	Psychocardiology	Unknown status; N/A	MUSE headband–based mindfulness training in patients with AF	Not available
	SMART program for paroxysmal atrial fibrillation	NCT03450993	Psychocardiology	Completed; N/A	Stress management and resilience training in patients with paroxysmal AF	The online SMART program improved coping and positive affect and reduced negative affect and AF‐related symptoms [328].
	The effect of a digital MediYoga program on quality of life in AF	NCT07321964	Psychocardiology	Recruiting; N/A	Digital MediYoga in patients with AF; assessment of QoL, stress, anxiety, and depression	Not available
	Internet‐based cognitive behavior therapy for atrial fibrillation	NCT02694276	Psychocardiology	Completed; N/A	Internet‐delivered CBT in patients with AF; assessment of symptom burden and QoL	Internet‐delivered CBT was feasible and potentially effective in patients with symptomatic AF [329].
	Internet‐delivered CBT compared with standard care for AF	NCT04962932	Psychocardiology	Completed; N/A	Internet‐delivered CBT in patients with AF; assessment of anxiety, depression, and QoL	Not available
	I‐CBT tailored to patients with cardiovascular disease and insomnia	NCT03938805	Psychocardiology	Completed; N/A	Internet‐delivered CBT for insomnia in patients with CVD	I‐CBT reduced Insomnia Severity Index scores [330].
	Behavioral activation and medication optimization in older adults undergoing cardiac procedures	NCT05575128	Psychocardiology	Completed; N/A	Behavioral activation and medication optimization for older adults undergoing cardiac procedures	Not available
Pharmacological therapy	BROKEN‐SWEDEHEART	NCT04666454	Takotsubo syndrome	Recruiting; Phase 4	Adenosine plus dipyridamole and apixaban; assessment of microvascular dysfunction and thromboembolic risk	Not available
	Cyclosporine in Takotsubo syndrome/CIT‐DZHK29	NCT05946772	Takotsubo syndrome	Recruiting; Phase 2	Cyclosporine A as an anti‐inflammatory therapy	Not available
	Verapamil as therapy for children and young adults With Dravet syndrome	NCT01607073	Epileptic heart	Completed; Phase 2	Verapamil as adjunctive therapy	Not available
	Fish oil [EPA and DHA] for epilepsy	NCT00871377	Epileptic heart	Completed; Phase 2	Omega‐3 fatty acids supplementation	Low‐dose fish oil supplementation reduced seizure frequency by 34% and lowered blood pressure [331].
	COgnitioN with VERiciGuat evaluation in heart failure/CONVERGE‐HF	NCT06601465	Cardiac dementia	Recruiting; Phase 2	Vericiguat in patients with chronic HF; assessment of cognitive and imaging biomarkers	Not available
	Oral carnitine in heart failure patients	NCT07201714	Cardiac dementia	Recruiting; early Phase 1	Oral carnitine in patients with HF; cognitive assessment using MoCA	Not available
	Cognitive impairment related to atrial fibrillation/GIRAF	NCT01994265	Cardiac dementia	Completed; N/A	Dabigatran versus warfarin in patients with AF; assessment of cognitive impairment	No difference in stroke, cognitive decline, or dementia was observed between dabigatran and warfarin groups over 2 years [332].
	Impact of anticoagulation therapy on cognitive decline and dementia in nonvalvular AF/CAF	NCT03061006	Cardiac dementia	Completed; Phase 4	Anticoagulation therapy in patients with nonvalvular AF; assessment of cognitive decline and dementia	Not available
	AntiCoagulants and COGnition/ACOG	NCT04073316	Cardiac dementia	Unknown status; Phase 4	Comparison of anticoagulation strategies in patients with nonvalvular AF; assessment of global cognitive performance	Not available
	Trial of apixaban vs warfarin in reducing rate of cognitive decline	NCT03839355	Cardiac dementia	Terminated; Phase 3	Apixaban versus warfarin in patients with AF; assessment of cognitive decline	Not available
	EMOTIon and COgNitive function after atrial fibrillation ablation/EMOTION‐AF	NCT04942171	Cardiac dementia	Unknown status; N/A	AF catheter ablation versus medical therapy; assessment of cognitive and emotional outcomes	Not available
	Canadian cardiac randomized evaluation of antidepressant and psychotherapy efficacy/CREATE	ISRCTN15858091	Psychocardiology	Completed; N/A	Citalopram and interpersonal psychotherapy in patients with CAD and major depressive disorder	Interpersonal psychotherapy provided no additional benefit in patients with CAD and major depressive disorder [333].
	Escitalopram in depressive patients with acute coronary syndrome	NCT00419471	Psychocardiology	Completed; Phase 4	Escitalopram in patients with depression after ACS	Escitalopram may improve long‐term cardiovascular outcomes in patients with depression after ACS [334].
	Antidepressant Medication Treatment for Depression in Chronic Heart Failure / SADHART‐CHF	NCT00078286	Psychocardiology	Completed; Phase 3	Sertraline in patients with HF and depression	Sertraline was well tolerated but did not improve depressive symptoms or cardiovascular status compared with placebo in patients with HF [335].
	Omega‐3 for treatment of depression in patients with heart failure/OCEAN	NCT02057406	Psychocardiology	Completed; Phase 3	Omega‐3 fatty acid supplementation in patients with chronic HF and major depressive disorder	Omega‐3 fatty acid supplementation improved cognitive depressive symptoms and social functioning [296].
	Omega‐3 for depression and other cardiac risk factors	NCT02021669	Psychocardiology	Completed; N/A	Omega‐3 fatty acid supplementation in patients with depression and cardiac risk factors	EPA plus sertraline was not superior to placebo plus sertraline [336].
	Omega‐3 polyunsaturated fatty acids on major depressive disorder in CVD	NCT03072823	Psychocardiology	Completed; N/A	n‐3 PUFA supplementation in patients with CVD and major depressive disorder	n‐3 PUFAs supplementation improved fatigue in patients with CVD and coexisting depression [337].

Abbreviations: ACS, acute coronary syndrome; AF, atrial fibrillation; AIS, acute ischemic stroke; BAT, baroreflex activation therapy; CAD, coronary artery disease; CAN, central autonomic network; CBT, cognitive behavioral therapy; CCT, computerized cognitive training; CMR, cardiac magnetic resonance; CVD, cardiovascular disease; ECG, electrocardiography; EEG, electroencephalography; GSN, greater splanchnic nerve; HF, heart failure; HFpEF, heart failure with preserved ejection fraction; HFrEF, heart failure with reduced ejection fraction; HRV, heart rate variability; LVEF, left ventricular ejection fraction; PCWP, pulmonary capillary wedge pressure; QoL, quality of life; SCS, spinal cord stimulation; SHS, stroke–heart syndrome; TTS, Takotsubo syndrome; VNS, vagus nerve stimulation; HIIT, high‐intensity interval training.

Specifically, trials of autonomic modulation include breathing training, drugs targeting the ANS, VNS, BAT, renal denervation, SCS, and GSN resection, blockade, or ablation. Biomarker monitoring mainly includes ECG‐based assessment of HR, HRV, and arrhythmias; EEG monitoring; and measurement of peripheral inflammatory and multiomics biomarkers. Neuroimaging, especially fMRI, is used for task‐based functional localization, functional connectivity analysis, and brain network analysis. Assessment of brain structural changes and cerebral blood flow may also help clarify the mechanisms underlying heart–brain interactions. Exercise‐related trials mainly focus on exercise modality, intensity, and training pattern, such as aerobic or resistance exercise, low‐, moderate‐, or high‐intensity training, and continuous or interval training. Psychological interventions mainly include CBT, cognitive training, behavioral activation, and mindfulness‐based interventions. Pharmacological trials mostly investigate the potential heart–brain benefits and effects on prognosis of existing drugs, including anticoagulants, antidepressants, anti‐inflammatory agents, and metabolic modulators.

Future clinical trials still require further refinement and methodological advances. For autonomic modulation, the key challenge is to identify which patterns of heart–brain dysregulation are most amenable to neuromodulation. For example, different HF phenotypes, including HFrEF, HFmrEF, HFpEF, and HFimpEF, may have distinct patterns of autonomic dysfunction. Therefore, future studies should further identify the optimal candidates, intervention timing, and individualized stimulation parameters. For biomarker monitoring, more sensitive and user‐friendly wearable electrophysiological devices and more accurate algorithms are needed. In particular, combined EEG–ECG monitoring may help capture abnormalities in heart–brain interactions in real time. Peripheral Aβ, inflammatory mediators, and multiomics biomarkers may also provide important insights into the pathological state of the heart–brain axis. Neuroimaging may be useful for screening, mechanistic investigation, and treatment‐response assessment in conditions involving heart–brain interactions. Its strength lies in detecting subtle changes in brain function and precisely localizing key brain regions. Psychological interventions may benefit from internet‐based platforms and smart devices, which could improve accessibility and adherence and support the long‐term management of psychocardiological conditions. Pharmacological therapy remains an important component of the management of conditions involving heart–brain interactions. Future studies should further clarify the key molecular mechanisms of heart–brain interactions, identify druggable targets, and facilitate the development of targeted therapies for heart–brain axis dysfunction.

## 5 Conclusion and Perspectives

### 5.1 Shared Mechanisms of Heart–Brain Interactions

Heart–brain interactions form a dynamic network shaped by neural regulation, biochemical signaling, and mechanical transduction. The neural pathway regulates cardiac electrophysiology and mechanical contraction through hierarchical connections among the CAN, ANS, ECNS, and ICNS. At the same time, afferent signals from the heart provide feedback to the CNS and influence autonomic activity, emotional responses, and cognitive processes. The biochemical pathway mediates heart–brain communication through circulating hormones, neuropeptides, inflammatory mediators, and metabolic signals. The mechanical pathway mainly relies on baroreceptors and mechanosensitive ion channels, such as PIEZO channels. These structures convert mechanical cues, including BP fluctuations, shear stress, cardiac pulsation, and respiratory motion, into biological signals, thereby contributing to coordinated feedback regulation between the heart and brain.

Under physiological conditions, these three pathways work together to coordinate cardiovascular and neural functions in response to changes in emotion state, stress, exercise, sleep, and hemodynamic conditions. In this way, they help maintain systemic homeostasis. In disease states, however, this normally adaptive regulatory network may become dysregulated. Sympathetic overactivation, amplified inflammation, abnormal mechanotransduction, and impaired heart–brain feedback regulation may reinforce one another, ultimately creating a vicious cycle of cross‐organ injury.

### 5.2 Disease‐Specific Manifestations and Shared Pathophysiological Loops

Although different diseases involving heart–brain interactions present with distinct clinical phenotypes, they often share similar pathological loops. TTS and the epileptic heart mainly reflect acute “brain‐to‐heart” effects. Intense emotional stress, abnormal cerebral electrical activity, or dysfunction of the CAN may rapidly trigger sympathetic overactivation, catecholamine release, and cardiac electrophysiological disturbances. These changes may ultimately contribute to myocardial stunning, arrhythmias, and an increased risk of sudden cardiac death. SHS represents another form of secondary cardiac injury after acute brain injury. This is particularly evident when stroke involves autonomic regulatory regions, such as the insula. In this setting, sympathetic activation, impaired baroreflex function, and systemic inflammation may contribute to myocardial injury, arrhythmia, and cardiac dysfunction.

Unlike acute “brain‐to‐heart” cardiac injury, HF and cardiac dementia are characterized by chronic bidirectional pathological cycles. HF progression is associated with sustained neurohumoral activation, particularly sympathetic activation, together with impaired baroreflex and chemoreflex function and enhanced inflammatory responses. Declining cardiac function may further aggravate central autonomic imbalance, creating a self‐amplifying process in which worsening HF is associated with progressively impaired neural regulation. The concept of cardiac dementia suggests that heart disease may contribute to cognitive decline and structural brain injury through cerebral hypoperfusion, cerebral microembolism, BBB disruption, neuroinflammation, and neurodegenerative changes.

Psychocardiological research further suggests that depression, anxiety, and chronic stress may be associated with increased cardiovascular risk through autonomic imbalance, HPA axis dysregulation, activation of inflammatory pathways, and abnormal interoception. Conversely, the symptom burden, perceived threat of death, and reduced quality of life associated with CVD may further exacerbate psychological disorders. Thus, although the six diseases categories have different initiating factors, they often converge on several shared mechanisms: autonomic imbalance, immune‐inflammatory activation, hemodynamic disturbances, brain network remodeling, and psychological or behavioral dysregulation. These processes reinforce one another and may form shared pathophysiological loops that contribute to the onset and progression of conditions involving heart–brain interactions.

### 5.3 Current Challenges and Future Directions: Toward Integrated Heart–Brain Medicine

Although research on heart–brain interactions is gradually moving from mechanistic exploration to clinical translation, several key bottlenecks remain. First, much of the current evidence is still derived from cross‐sectional studies, retrospective cohort studies, or animal models. These studies can suggest associations between cardiac and neurological conditions, but they are not sufficient to establish causality or define the temporal sequence of pathological events. Second, diseases involving heart–brain interactions still lack a unified system for mechanism‐based classification and risk stratification. Although different diseases may share pathophysiological processes, such as autonomic imbalance, immune‐inflammatory activation, cerebral hemodynamic abnormalities, arrhythmia, and psychological or behavioral abnormalities, the relative importance of these mechanisms may vary across disease stages and patient groups. Therefore, classification based solely on disease labels may not accurately reflect the main pathological drivers in individual patients or effectively guide precision interventions. Future studies should develop mechanism‐based classification frameworks to identify key pathophysiological pathways, high‐risk patients, and key therapeutic targets.

Another major challenge is the lack of a mature dynamic and multimodal monitoring system. Heart–brain interactions are dynamic processes involving synchronized changes in brain electrical activity, cardiac rhythm, fluctuations in autonomic activity, cerebral blood flow, inflammatory activity, metabolic alterations, and psychological or behavioral responses. However, most existing studies still rely on single‐time‐point or unimodal measurements. These approaches are insufficient to capture real‐time interactions between the heart and brain. Future studies should further integrate EEG–ECG monitoring, wearable devices, neuroimaging, peripheral blood multiomics, and artificial intelligence algorithms. Such integration may help establish reproducible and scalable systems for risk prediction and treatment response assessment. Such systems could support early identification of high‐risk patients and enable more precise stratification for neuromodulation, psychological interventions, exercise therapy, and pharmacological treatment.

At the therapeutic level, existing clinical trials have covered neuromodulation, exercise training, psychological interventions, and pharmacological therapy. However, the overall evidence remains insufficient. Many studies are limited by small sample sizes, heterogeneous outcome measures, short follow‐up periods, and a lack of mechanism‐based stratification. For neuromodulation, the optimal candidates, stimulation targets, intervention timing, and individualized stimulation parameters remain to be defined. Psychological interventions and exercise therapy are generally considered safe and accessible, but their long‐term effects on heart–brain outcomes require further evaluation in larger studies. Pharmacological research still relies mainly on the repurposing of existing drugs, and few novel targets based on key mechanisms of the heart–brain axis remain have been identified.

Future heart–brain medicine should move beyond single‐organ treatment toward integrated management. A coherent research framework is needed to integrate mechanism identification, risk stratification, dynamic monitoring, and precision interventions. The goal is to identify heart–brain axis vulnerability at an earlier stage and prevent or slow the progression of cross‐organ injury through individualized treatment.

## Author Contributions

W.W. and H.H. were involved in writing – review and editing and writing – original draft. H.J. was responsible for supervision. J.S. contributed to project administration and funding acquisition. All authors read and approved the final manuscript.

## Funding

This work was supported by the National Natural Science Fund for Distinguished Young Scholars of China (Grant No. 82125004) and the Fundamental Research Funds for the Central Universities, Peking Union Medical College (Grant No. 3332025130).

## Ethics Statement

The authors have nothing to report.

## Conflicts of Interest

The authors declare no conflicts of interest.

## Data Availability

The authors have nothing to report.
